# Transcriptomic profile of cystic fibrosis patients identifies type I interferon response and ribosomal stalk proteins as potential modifiers of disease severity

**DOI:** 10.1371/journal.pone.0183526

**Published:** 2017-08-28

**Authors:** Michael S. D. Kormann, Alexander Dewerth, Felizitas Eichner, Praveen Baskaran, Andreas Hector, Nicolas Regamey, Dominik Hartl, Rupert Handgretinger, Justin S. Antony

**Affiliations:** 1 Department of Pediatrics I, Pediatric Infectiology and Immunology, Translational Genomics and Gene Therapy in Pediatrics, University of Tuebingen, Tuebingen, Germany; 2 Center for Quantitative Biology, University of Tuebingen, Tuebingen, Germany; 3 University Children's Clinic Department of Paediatrics I, Paediatric Infectiology & Immunology, University of Tuebingen, Tuebingen, Germany; 4 Division of Paediatric Respiratory Medicine and Department of Clinical Research, University of Bern, Bern, Switzerland; 5 Children’s Hopsital of Lucerne, Paediatric Pulmonology, Lucerne, Switzerland; 6 University Children's Clinic Department of Paediatrics I, Hematology and Oncology, University of Tuebingen, Tuebingen, Germany; Lee Kong Chian School of Medicine, SINGAPORE

## Abstract

Cystic Fibrosis (CF) is the most common monogenic disease among people of Western European descent and caused by mutations in the *CFTR* gene. However, the disease severity is immensely variable even among patients with similar *CFTR* mutations due to the possible effect of ‘modifier genes’. To identify genetic modifiers, we applied RNA-seq based transcriptomic analyses in CF patients with a mild and severe lung phenotype. Global gene expression and enrichment analyses revealed that genes of the type I interferon response and ribosomal stalk proteins are potential modifiers of CF related lung dysfunction. The results provide a new set of CF modifier genes with possible implications as new therapeutic targets for the treatment of CF.

## Introduction

Cystic fibrosis (CF) is the most common life-threatening genetic disease among people of Western European descent. The disease is caused by a mutation in the underlying disease-conferring gene, Cystic Fibrosis Transmembrane and Conductance Regulator (*CFTR*) that encodes a chloride channel. CFTR mutations result in disrupted chloride transport in the epithelial cells of various organs including lung, intestine, pancreas, and testes [1]. More than 2000 *CFTR* genetic variants have been reported and categorized into six classes, as these mutations were observed to exhibit diverse effects on the production of CFTR protein, its trafficking, its function, and its stability at the epithelial cell membrane [1, 2]. The most prevalent mutation in CF is the lack of a phenylalanine residue at position 508 (also known as F508del), which results in a misfolded CFTR protein with reduced transmembrane conductance of chloride ions [3]. Due to the trafficking defect, the F508del mutation prevents the CFTR protein reaching the apical membrane of epithelial cells and also interrupts the normal movement of chloride ions by disrupting CFTR channel gating [4].

Chronic progressive lung disease is the primary reason for morbidity and mortality in CF. However, the disease severity is immensely variable even among patients with similar CFTR mutations. For instance, CF patients with a homozygous F508del mutation showed varying phenotypic heterogeneity in terms of lung function that is measured by forced expiratory volume in 1 second (FEV_1_) [5]. It has been observed that *CFTR* genotypes *per se* do not corroborate with the pulmonary phenotype in unrelated CF patients [6]. However, twin and sibling studies clarified that the modifier genes and their genetics contributed substantially to divergent outcomes observed in CF lung disease [7, 8]. As CF is a multi-organ disease, the contribution of modifier genes towards disease severity was relatively higher when compared to non-genetic modifiers in specific organs [9].

Modifier genes are a vital part of human genetics and it is argued that they are responsible for the diverse phenotype observed with various diseases, more precisely in CF [10, 11]. Several CF related modifier genes have been reported through genome-wide association studies (GWAS), hypothesis-driven candidate gene studies, and microarray based transcriptomic analyses [5, 9, 12–15]. Unlike GWAS and candidate gene studies, transcriptomic analyses provide information on both genetic modifiers and non-genetic modifiers such as infections from the environment. The host immune system responds to these infections by activating immune genes, and these gene transcripts can be measured by transcriptomic approaches. Though microarray-based transcriptomic analyses could identify some modifier genes, the technology is limited to known transcripts [12, 16, 17]. However, global gene expression analysis using RNA-seq provides several advantages over microarray including the detection of a higher number of differentially expressed genes and the detection of novel transcripts [18]. Therefore, in the present study we performed a transcriptomic analysis using RNA-seq on CF patients with an identical genotype (homozygous F508del), but with mild and severe lung dysfunction determined by FEV_1_ measurements.

## Materials and methods

### Patient samples

A total of 32 F508del homozygous CF patients were recruited for the study from the University Childrens’ Hospital, Tuebingen, Germany and the Division of Paediatric Respiratory Medicine, University of Bern, Switzerland. All blood samples were collected in PaxGene blood vacutainer tubes to ensure RNA quality and significantly reduce RNA degradation. Participants were classified into severe CF patients (n = 16) and mild CF patients (n = 16) based on their FEV_1_ values, which is a standard to quantify the clinical phenotype of CF. The detailed clinical characteristics of the enrolled patients are described in Table 1. For statistical analyses of clinical parameters, data were analyzed using the Mann-Whitney U test and are presented as means and SDs, unless stated otherwise. Ethical approval was obtained from the Institutional review board, Children’s University Hospital Tuebingen, Germany. Informed written consent was obtained from all participants; for those who were children, consent was obtained from respective parents or guardians.

**Table 1:** Clinical characteristics of the mild CF and severe CF samples.

Clinical Parameters	Mild CF patients	Severe CF patients	*P* value
Number (n)	16	16	Not Significant
Age, mean (SD)	25 (12)	21 (9)	Not Significant
Male:Female	10:6	8:8	Not Significant
CFTR Mutation	F508del/ F508del	F508del/F508del	Not Significant
FEV_1_ (% predicted), mean (SD)	92 (16)	36 (10)	< 0.0001***
Infections	PSA, SA, CA, SM	PSA, SA, CA, SM, AF	Not Significant

*** Mann-Whitney *U* test.

PSA, *Pseudomonas aeruginosa*; SA, *Staphylococcus aureus*; CA, *Candida albicans*; SM, *Stenotrophomonas maltophilia*; AF, *Aspergillus fumigatus*.

### RNA library preparation

Total RNA was isolated with PAXgene tubes using a Qiacube roboter with standard protocols (www.qiagen.com). Excess globin mRNA was removed with GLOBINclearTM kit and sequencing libraries were prepared with the TruSeqTM Total RNA Sample Prep Kit following the manufacturer’s instructions (www.illumina.com). Each sequencing library was tagged with a 6 nucleotide long barcode that identifies from which sample a sequence was derived. After size selection, all sequencing libraries were quantified on a Qubit® fluorometer and equimolar amounts of 16 libraries were combined to generate two sequencing pools. These were loaded onto 8 lanes of an Illumina GAIIx single-read flow cell and two MiSeq flow cells. Bound molecules were clonally amplified on a cBot instrument. Subsequently, the first 50 nucleotides from each fragment were sequenced followed by a seven nucleotide sequencing run to decipher the barcode sequence in the adapter (www.illumina.com).

### RNA-seq data analysis

The quality of raw sequenced reads was assessed using the fastqc quality control tool and high-quality reads were aligned against the *Homo sapiens* reference genome (hg19) using the STAR RNA-seq alignment tool [19]. The STAR aligners automatically detect and remove adaptor sequences from the sequence reads before alignment. Next, we calculated the total number of mapped reads and the number of uniquely mapped reads for each sample. As an additional layer of quality control, we discarded samples with low mapped read count and samples with a small percentage of uniquely mapped reads. For samples that pass the additional quality check, we estimated the gene expression level as CPM (count per million mapped reads) using HTSeq [20]. We used EdgeR R package to identify differentially expressed genes (DEGs) between mild CF and severe CF [21]. RNA-Seq reads are available in NCBI-Sequence Read Archive (SRA) database, under accession number SRP111640. Samples in each CF group were treated as biological replicates and only genes that pass the significance cut-off were treated as DEGs. In this study, we used log fold-change in expression less than -0.5 or greater than 0.5 and *P*-value (FDR corrected *P*-value) less than 0.05 as the significance cut-off to identify DEGs. In order to functionally categorize DEGs, we tested enrichment of Gene Ontology (GO) terms among DEGs using Amino.2 (www.amigo.geneontology.org). Additionally, we also analyzed DEGs for the over-representation of KEGG pathways (http://www.genome.jp/kegg/pathway.html). Motif activity response analysis (MARA) was performed to predict the global regulatory interaction of RNA-seq data using ISMARA online tool (www.ismara.unibas.ch). MARA predicts transcription factor (TF) binding sites with a specific algorithm and calculates the influence of specific TF in terms of gene expression in a given sample. Mann-Whitney U rank sum tests were applied to analyze differences in motif activity between the study groups. All the major DEGs were checked for their association with CF using Open target Platform (www.targetvalidation.org).

### qPCR validation

qRT-PCR validation was performed for 16 of the DEGs utilizing the same samples utilized for RNA-seq as a technical reproducibility operating different platforms. Primers were designed with the Primer3 online tool (www.primer3.ut.ee) Primers, amplicon details, and cycling conditions are listed in Table 2. cDNA was synthesized using iScript (www.biorad.com), followed by qRT-PCR using Power SYBR green, and samples were run in triplicate on a ViiA7 Real-Time PCR System (www.lifetechnologies.com). The relative expression levels of selected DEGs were normalized to the 18S rRNA. Differences in mRNA expression between mild CF and severe CF were analyzed by pair-wise fixed reallocation randomization tests with REST 2009 software [22]. The severe CF patients group was used as a control group and statistical significance was determined using randomization tests. In addition, the expression of each gene was determined for every single patient using the 2^-ΔΔCt^ method and Mann-Whitney U tests were applied for separate gene analyses between study groups. The level of significance was set to a *P*-value of <0.05.

**Table 2:** Primer details and PCR cycling conditions for the qPCR validation.

Gene	Primer sequences (5'-3')	Amplicon Size (bp)	Annealing Temperature (°C)
HERC5	F: CCAGCTTGCTTGTCCAACAG	157	58
	R: CGGCCAGTAAACCCTCTTCT		
IFIT1	F: TGGACCCTGAAAACCCTGAA	243	54
	R: TCTGTGAGGACATGTTGGCT		
IFIT2	F: GCGAAACAACTGCTCCATCT	205	55
	F: CCAAGACATGCAAAGCCTCA		
RSAD2	F: AAGAGGAGGAAGAGGACCCT	250	55
	R: CAGAACCTCACCAACTTGCC		
IFI44L	F: GAGCAACTGGTGTGTCGTTT	213	56
	F: CCTATTTCTGTGCTCTCTGGC		
GOS2	F: GGAATGGAGAGACAGAGGGG	239	59
	R: AGTGCAAAATGGTAGACGCA		
CXCL10	F: TGGATGTTCTGACCCTGCTT	201	56
	R: AAAGAATTTGGGCCCCTTGG		
FOSB	F: TCTGTCTTCGGTGGACTCCTTC	209	57
	R: GCAAACCGTAGATGCTCAGGG		
OAS3	F: GCTTCACAGAGCTACAACGG	167	58
	R: CTCCCAGGCATACACAGTCA		
IFI6	F: AGCAGCGTCGTCATAGGTAA	213	56
	R: TGCACTCTAGCCTGGACAAT		
MX1	F: CATCCAGCCACCATTCCAAG	168	57
	R: AGAATCGCTTGAACCTGGGA		
EGR1	F: AGCTGGAGGAGATGATGCTG	257	54
	R: CCAGCACCTTCTCGTTGTTC		
IL8	F: TCTTGGCAGCCTTCCTGATT	211	56
	R: TCCAGACAGAGCTCTCTTCCATC		
LOC644172	F: CCGACGTCCATTTCTCCAAG	184	58
	R: TCATCCACTTCCAGCTCAGG		
ZFN683	F: AGCCTTGCCTTACCCGCTGAAA	129	60
	R: AATGGACGCTCTCCACTGTGCA		
EPB41L4B	F: ACCCACTTCCTTGACAGAGT	233	55
	R: CGCAAGTTAGCAGCACCAAT		
18s rRNA	F: GTAACCCGTTGAACCCCATT	151	54
	R: CCATCCAATCGGTAGTAGCG		

## Results

We investigated the transcriptomic profile of peripheral blood leukocytes from mild and severe CF patients using RNA-sequencing. No differences were evident for important clinical parameters between the investigated groups, except for the FEV_1_ status (*P*<0.0001; Table 1). After the quality check for RNA-seq data, three samples were removed from downstream analyses due to the low number of total reads and lower mapping rate (S1 Fig). In total, 12,778 genes with at least ten reads per sample were included in the final analysis stage. The principal component analysis (PCA) of the final data revealed that the differences in the expression profiles of the two investigated groups were relatively small (S2 Fig).

### Differentially expressed genes (DEGs) between mild CF and severe CF

**Fig 1:**
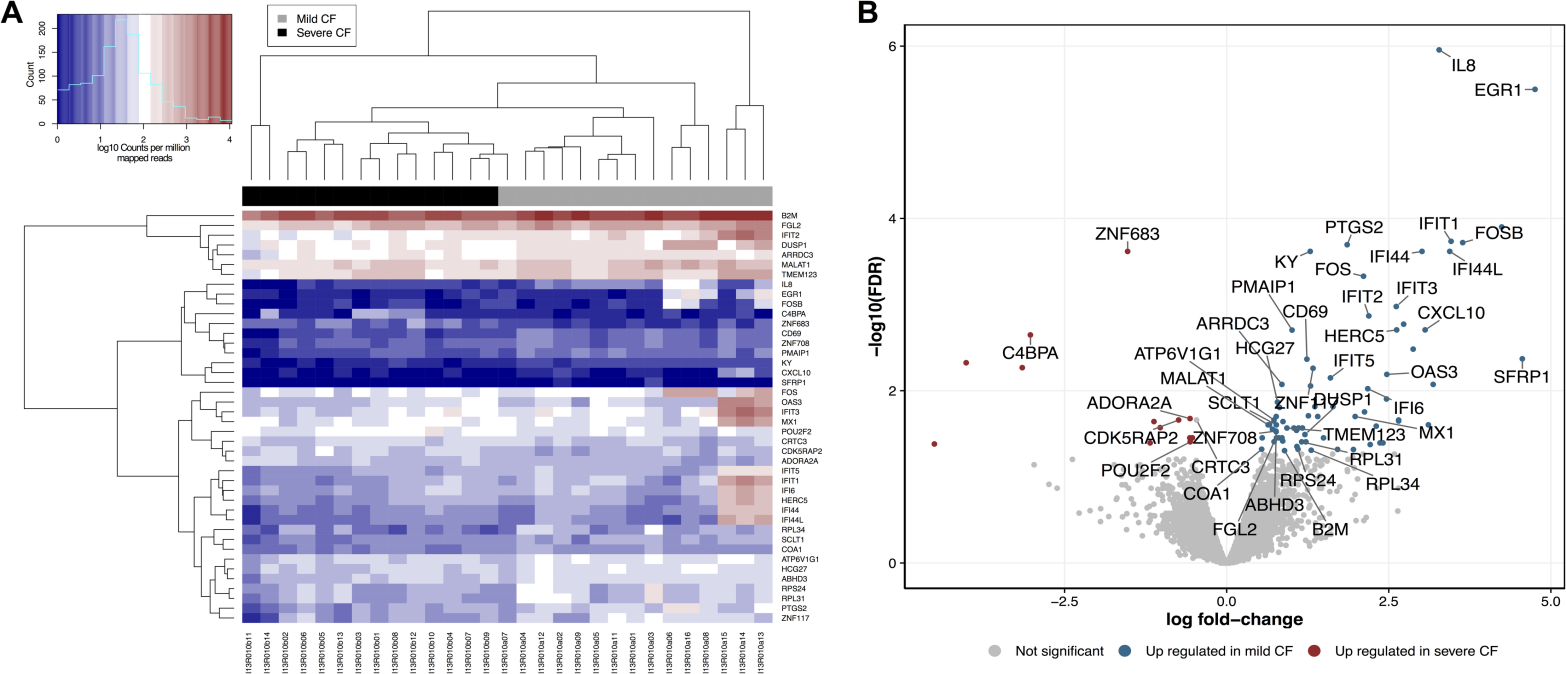
RNA-seq analyses of Mild CF and severe CF patients. **A)** Heat-map of differentially expressed genes. Each column represents a separate patient, and each horizontal line represents a separate gene. Dendrogram of clustered samples and genes, in which mild CF and severe CF samples cluster with respect to their expression similarity. Expression profiles are measured by counts per million reads (CPM- model). **B)** Volcano plot of RNA-seq data, in which the -Log_10_ of the false discovery rate is plotted against Log_2_ fold change.

Differential expression analysis showed that 88 genes were differentially expressed between mild and severe CF group of patients, among which 74 genes (84%) had higher expression levels in mild CF patients and 14 (16%) genes had higher expression levels in individuals with severe CF. The results from this analysis with respective expression levels and adjusted *P*-values can be found in S1 Table. We compared DEGs between mild and severe CF patients and the heat-map of the selected DEGs revealed two distinct clusters for the investigated groups (Fig 1A). Global gene level expression analysis between the groups (mild CF vs. severe CF) is shown in volcano plots (Fig 1B) with respective gene name, expression level and accuracy of the detection using RNA-seq. *EGR1*, *SFRP1*, *RSAD2*, and *FOSB* are the major DEGs that were significantly overexpressed (>3.5) in the mild CF group in comparison with the severe CF group. Increased expression of *EGR1* was found to be significantly associated with mild lung disease (fold change = 4.5; FDR *P* = 3.1x10^-6^). Concordantly, other EGR family genes, *EGR2* (fold change = 2.4; FDR *P* = 0.04) and *EGR3* (fold change = 2.6; FDR *P* = 0.02) showed higher level of expression in the mild CF group. Similarly, *EPB41L4B*, *LOC644172*, *C4BPA*, and *ZNF683* are the major DEGs that were significantly overexpressed (>1.5) in the severe CF group (S1 Table). Among these genes, *ZNF683* exhibited better association with the severe form of CF lung disease (fold change = 1.5; FDR *P* = 0.0002).

### qPCR validation of RNA-seq data

**Fig 2:**
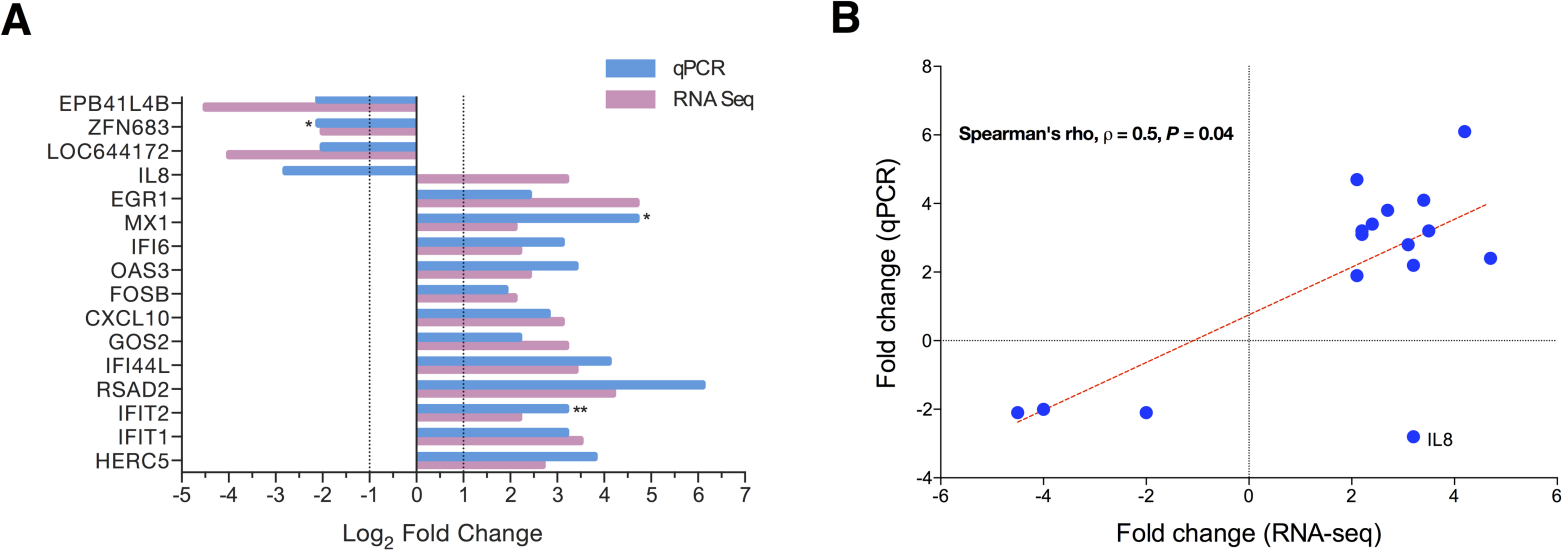
qPCR validation of RNA-seq findings. **A)** Expression profile of RNA-seq data and qPCR data for selected genes were compared using the same samples. Severe CF samples were used as control group and the expression level set to 1 or -1. Expression was normalized using *18s* as a reference gene. Results represent mean values and are expressed as Log_2_ values of the fold change. Significant difference observed in qPCR results between mild CF and severe CF patients (**P value < 0.05; ***P value < 0.001). The level of significance was set to a *P*-value of < 0.0001 for RNA-seq data. **B)** Correlation analysis of RNA-seq and qPCR. (Log_2_ values of the fold change; Spearman’s rho, ρ = 0.53, *P* value = 0.04).

**Fig 3:**
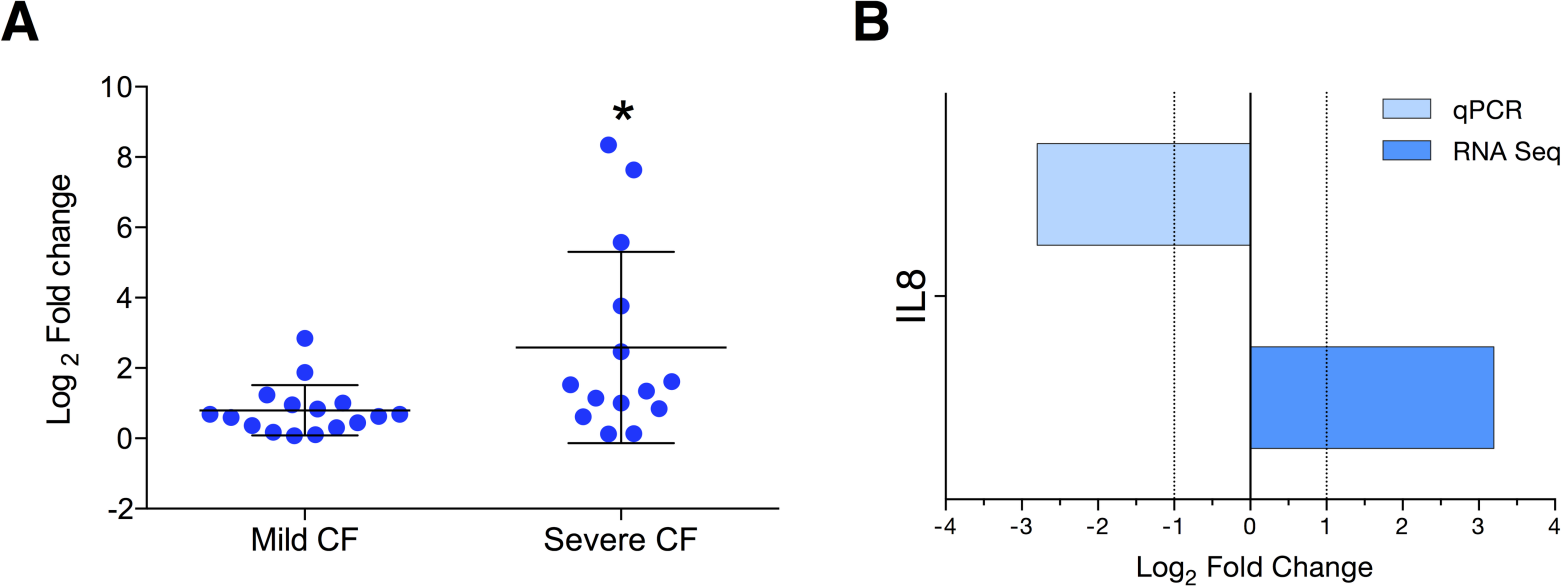
IL-8 expression by qPCR and RNA seq. **A**) Dot-plot shows the expression of IL-8 in severe and mild CF patients. Expression was normalized using *18s* as a reference gene. Results represent mean values and are expressed as Log_2_ values of the fold change. **B**) Using the same data of Fig 2A for IL-8 to understand different results between platforms.

We validated RNA-seq findings for 16 DEGs by qPCR using the same samples and observed high concordant results between RNA-seq and qPCR (94%; 15 out of 16 DEGs were verified; Fig 2A). The comparison of DEG expression levels between RNA seq versus qPCR revealed better correlation (Spearman’s rho, ρ = 0.53, *P =* 0.04; Fig 2B). Thus, these 15 genes had similar mRNA levels, as RNA-Seq and qRT-PCR data were comparable. Furthermore, significant difference in expression levels was observed in qPCR results for the mild CF group with following genes, *IFIT2* (fold change = 3.2, *P<*0.001), *MX1* (fold change = 3.2, *P<*0.05) and *ZFN683* (fold change = -2.1, *P<*0.05). No significant difference was achieved for *EGR1* as many samples of the severe CF group failed in amplification (data not shown). IL-8 was the only DEG that could not be verified by qPCR. Since IL-8 is a very important proinflammatory cytokine in CF settings, we further dissected the IL-8 data for both platforms. RNA-seq data showed that IL-8 was overexpressed in the mild CF group (Figs 2A and 3B). Conversely IL-8 was significantly overexpressed in the severe CF group according to qPCR analysis (*P* = 0.01; Fig 3A).

### Enrichment and MARA analysis

**Fig 4:**
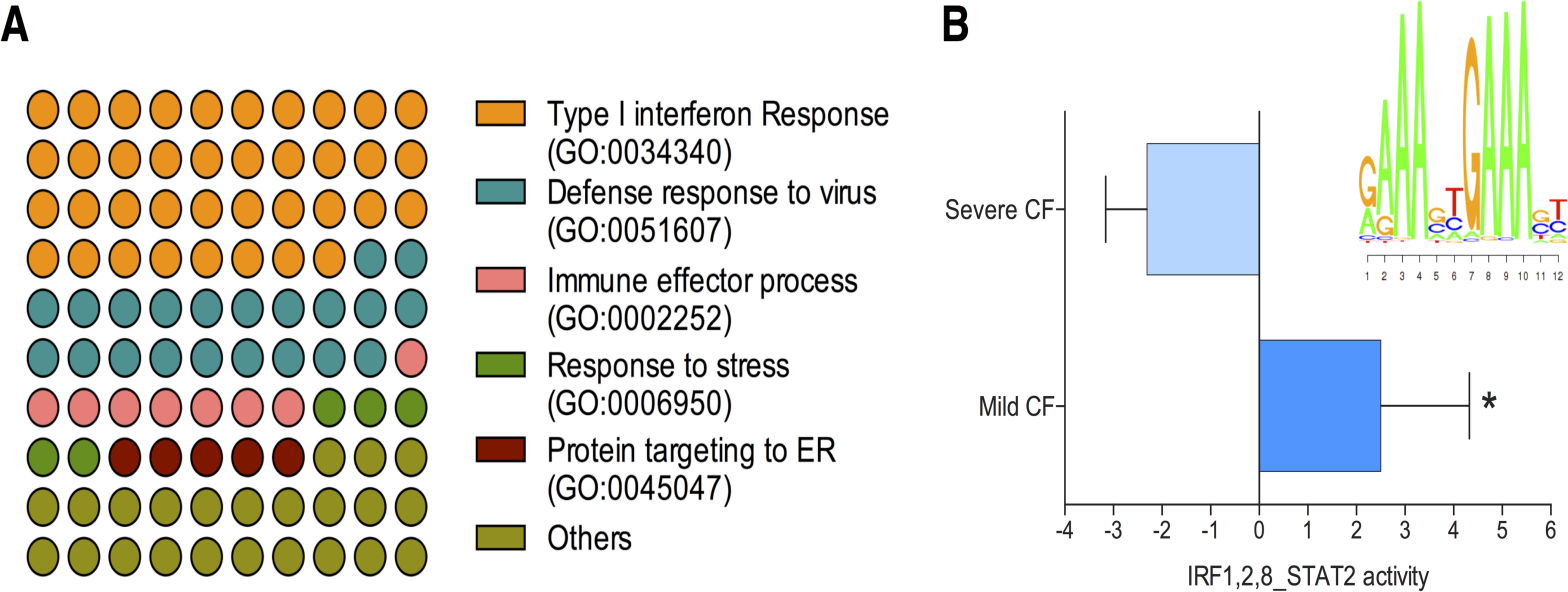
Functional enrichment analysis of RNA-seq data. **A)** Representative Gene Ontology (GO) terms enrichment among differentially expressed genes for biological processes. Genes involved in Type I interferon response, and ribosomal proteins responsible for endoplasmic reticulum transport and protein synthesis were over represented. **B)** ISMARA analysis predicts significant difference in IRF1,2,8 and STAT2 activity between mild CF and severe CF patients (*P value < 0.05).

To understand the biological processes that could be modified differently between the mild CF and the severe CF group, we performed a GO enrichment analysis for DEGs that are upregulated in mild and severe CF. In case of the mild CF group, genes that belongs to type I interferon response were selectively enriched (38%, *P* = 1.45E-09; Fig 4A, S2 Table). A total of ten genes involved in this pathway showed elevated expression in the mild CF group (*EGR1*, *MX1*, *IFIT1*, *IFIT2*, *IFIT3*, *OAS3*, *OASL*, *IFI6*, *ISG15*, and *RSAD2*). In addition, the pathways of the anti-viral response and the immune effector process showed significant enrichment (S2 Table). Enrichment analysis identified three ribosomal stalk protein genes *RPL31*, *RPL34*, and *RPS24* were enriched in the pathway of protein targeting to the endoplasmic reticulum (ER). No specific pathway was enriched for the fourteen-upregulated genes of the severe CF group. To explore the contribution of specific TFs and regulatory networks of DEGs we implemented MARA analysis to our RNA-seq data. Our data revealed that *IRF1*, *IRF2*, *IRF8*,and *STAT2* transcription factor activity were significantly increased in the mild CF group (mean activation z-score for mild CF 2.5, mean activation z-score for severe CF -2.3; *P* < 0.05; Fig 4B) with elevated transcript levels of their regulated genes. Top target genes (Target score >10) for these TFs belong to the type I interferon response pathway. The highest score (Target score >25) was observed for the following genes: *RSAD2*, *IFI44L*, *IFIT1*, *ISG15*, *IFIT3*, *OAS3*, *HERC5*, and *IFI44* (S1 File). Both our enrichment and pathway analysis were consistent in identifying the possible contribution of the type I interferon response in the mild CF phenotype. The network analysis of *IRF1*,*2*,*8* and *STAT2* transcription factors showed closer interactions of the above mentioned genes (S3 Fig) and other related genes in the type I interferon response.

## Discussion

CF patients with identical inherited mutations in the *CFTR* locus exhibit substantial variation in disease severity and lung function. The phenotypic heterogeneity of CF is majorly affected by modifier genes [23]. Though several approaches are utilized to identify possible modifier genes of CF, here we used a transcriptomics approach for this purpose [11]. A previous study by Wright *et al*., attempted to identify modifier genes between mild and severe CF patients in their nasal respiratory epithelial cells by a transcriptomics approach using microarray technology. However global gene expression analysis using RNA-seq has many advantages over microarray [24]. Therefore, in this study we primarily used RNA-seq technology to identify modifier gene(s) of mild and severe lung phenotype in CF patients homozygous for the F508del mutation. Primarily, we assessed whether our transcriptomics results from peripheral blood leukocytes were comparable to native cells of the respiratory system (nasal, tracheal, and bronchial epithelial cells) to avoid possible difference in these cell types. We compared our data with six different studies and observed similar expression patterns for genes including *CXCL10*, *GOS2*, *PTGS2*, *IFIT1*, *IFIT3*, and *ISG15* [12, 16, 17, 25–27]. Thus, upregulated gene profiles are relatively similar between blood leukocytes and native respiratory cells in CF. Likewise, both RNA-seq and microarray platforms resulted in common upregulated genes. Our data suggest that the blood cell transcriptome can be used as a surrogate for both upper and lower airway respiratory cells in CF. In addition, our RNA-seq data were validated using qPCR with the same samples and resulted in a high coherence score (94%).

The differences in the expression profiles of the two investigated groups are relatively small as they resulted in only 88 DEGs. However, we observed striking differences for these genes between the mild and severe pulmonary phenotype of CF. Seventy four genes exhibited higher expression in patients with mild CF lung disease compared with severe CF lung disease. A total of 14 genes showed a significant upregulation in individuals with severe CF compared with those with mild CF. Interestingly, enrichment analysis revealed that genes involved in the type I interferon response and the defense response to viral infections were highly enriched in the mild CF group. Though previous studies reported the association of type I interferon genes (*IFIT1*, *IFIT3*, and *ISG15*) with CF [12], the present study confirms the role of the type I IFN pathway in modifying CF. A very recent study using a systems biology approach identified a type I interferon gene *IFI16*, as a major CF modifier gene that alters lung function [28]. The type I IFNs are well-known for anti-viral response, and viral infections are potential contributors for a decline in lung function in CF patients [29]. Therefore, we carefully considered the influence of viral infections with the observed expression profile. However, all our study participants did not exhibit symptoms of cold or upper respiratory viral infections during blood sampling. Therefore, we deemed that the contribution of viral infections to the observed DEGs between mild and severe CF patients is less likely. However, increasing evidences suggest that Type I IFN signaling pathway is involved in host defense against other pathogens including bacteria, parasite and fungi [30]. For example, the type I interferon gene expression and their polymorphisms were prominently associated with susceptibility to systemic candidiasis [31]. Notably, earlier studies confirmed that the type I interferon signaling was activated by *Pseudomonas aeruginosa* in normal lung epithelial cells but was abrogated in CF epithelial cells [32]. *P*. *aeruginosa* is a well-known pathogen in CF patients that has been directly associated with decline in pulmonary function [33]. Of note, our study patients in both groups had fungal and bacterial infections without significant differences in their distribution between groups. Therefore, it is rational to hypothesize that due to the enhanced Type I IFN signaling, mild CF individuals kept their infections and lung function decline in control. This hypothesis can be further supported by the anti-bacterial effect of the type I IFN response in lung epithelium and the respiratory tract [34, 35]. The effect of another interferon related gene, *IFRD1*, in modifying the pathogenesis of CF is well studied by others and us [15, 36]. Together, our findings support the contribution of IFN, specifically, the type I IFN response in modifying lung function associated with CF.

Among the upregulated genes of the type I IFN response, *EGR1* was a prominent gene that shows higher expression in mild CF patients. A previous transcriptomic study had identified that *EGR1* was downregulated in *P*. *aeruginosa* infected CF bronchial epithelial cells [37]. Besides, *EGR1* was observed to be upregulated in *A*. *fumigatus* and *Toxoplasma gondii* infected cells [38], which signifies its significance during infections. Interestingly, we found that two other EGR family members, *EGR2* and *EGR3*, were also upregulated in the mild CF group. EGRs are a family of DNA-binding zinc-finger proteins and function as transcriptional regulators. In addition, EGR proteins were found to closely interact with other type I IFN genes including *Mx1*, *HERC5*, *RSAD*, and others. Thereby, the role of EGRs as potential modifier genes in CF lung disease warrants future research. Other potential modifier genes in type I IFN signaling are *Mx1* and *IFIT2*, as they play a dominant role against *P*. *aeruginosa* and their expression also was verified by qPCR [35]. MARA analysis resulted in higher transcriptional activity of IRF1,2,8 and STAT2 TF in mild CF patients and they are responsible for the higher expression of Type I IFN genes. Enrichment analysis identified three ribosomal stalk proteins *RPL31*, *RPL34* and *RPS24*, that were highly expressed in individuals with mild CF. These proteins were observed to be involved in protein trafficking to the ER and golgi transport. The relevance of ribosomal stalk proteins in CF is well documented. Earlier study in primary human bronchial epithelial cells with a homozygous F508del mutation showed that silencing of *RPL12* was rescued by CFTR ion channel activity [39]. In addition, another ribosomal stalk protein RPL27 was reported as chloride-dependent gene, which expression is related to the function of the CFTR channel [40]. As the F508del mutation is related to protein trafficking and early degradation in ER, these ribosomal stalk proteins are attractive for further research.

The CF lung environment is dominated by neutrophils and elevated levels of the proinflammatory chemokine, IL-8 [41]. A recent study presented a genetic association of IL-8 polymorphisms with FEV_1_ status of CF patients [42]. In our study, we observed increased expression of IL-8 transcripts in individuals with mild CF. However, this observation could not be verified by qPCR as more IL-8 expression was found in the severe CF group. A similar results was observed in nasal epithelia cells between mild and severe CF and argued that IL-8 is not predictably associated with severity of the disease [17]. However, as we noted a contradicting result between RNA-seq and qPCR, further validation with independent samples seems essential to explore the role of IL-8 in CF lung function variability. GWAS meta-analysis study with a high sample number (n = 6,365) revealed the strong association of five genomic regions to CF lung disease severity [13]. We examined whether any of the observed DEG shares the specified genomic location. Remarkably, we found that chromosomal mapping has assigned the *HCG27* gene to position 6p21.33, which is very close to the reported *HLA-DRA* locus at 6p21.32. Although *HCG27* is reported to be a pseudo-gene, we observed a significant difference in expression levels between mild and severe CF individuals and the region including *HCG27* is also a major susceptibility loci for psoriasis [43]. Nevertheless, our present study using a RNA-seq based transcriptomics approach was able to locate reported susceptibility loci and thereby replicate the larger GWAS study. Although other genes described earlier do not exactly match those identified in our study, they belong to the same biological families. For example, *ATP12A* was reported to be responsible for airway acidification in CF, and we identified *ATP6V1G1*, *a gene of the same family*, *in our study* [44].

Collectively, our comparative transcriptomic analysis between mild and severe CF lung disease provided new insights into CF pulmonary decline. The global gene expression analyses identified that genes of the type I interferon response and ribosomal stalk proteins and revealed potential CF modifier genes. Our findings have the potential for picking new therapeutic targets from from this list of genes for treatment of cystic fibrosis.

## Supporting information

S1 Fig Quality control of RNA-seq data. Absolute (A) and relative (B) number of reads mapped to hg19.
(TIFF)

S2 Fig Principal component analysis of mild and severe CF patients. PCA of the filtered CPM counts data to assess the differences in the expression profiles of the two investigated groups. Each point represents one sample; the closer two points are the more similar are the expression profiles. CF manifestation is color-coded.
(TIFF)

S3 Fig Network analysis of RNA-seq data. Network of MARA analysis predicts the possible interaction of IRF1,2,8 and STAT2 target genes.
(TIFF)

S1 Table List of DEGs in mild and severe CF patients. The results of differential expression analysis between mild and severe CF group. The respective expression levels given as a fold change and CPM with corrected *P*-Values.
(PDF)

S2 Table GO Enrichement analysis of RNA-seq data. The results of GO enrichement analysis for DEGs that are upregulated in mild and severe CF. Values of fold enrichment of specififed pathways with with corrected *P*-values were stated.
(PDF)

S1 File MARA analysis of RNA-seq data. The complete raw data of MARA analysis that predicted the IRF1,2,8 and STAT2 activity in RNA-seq.
(PDF)
