# Supplementary figures and images for: Transcriptomic profile of cystic fibrosis patients identifies type I interferon response and ribosomal stalk proteins as potential modifiers of disease severity
Source: PLoS One. 2017 Aug 28;12(8):e0183526. doi: 10.1371/journal.pone.0183526 (PMC5573219; doi:10.1371/journal.pone.0183526)

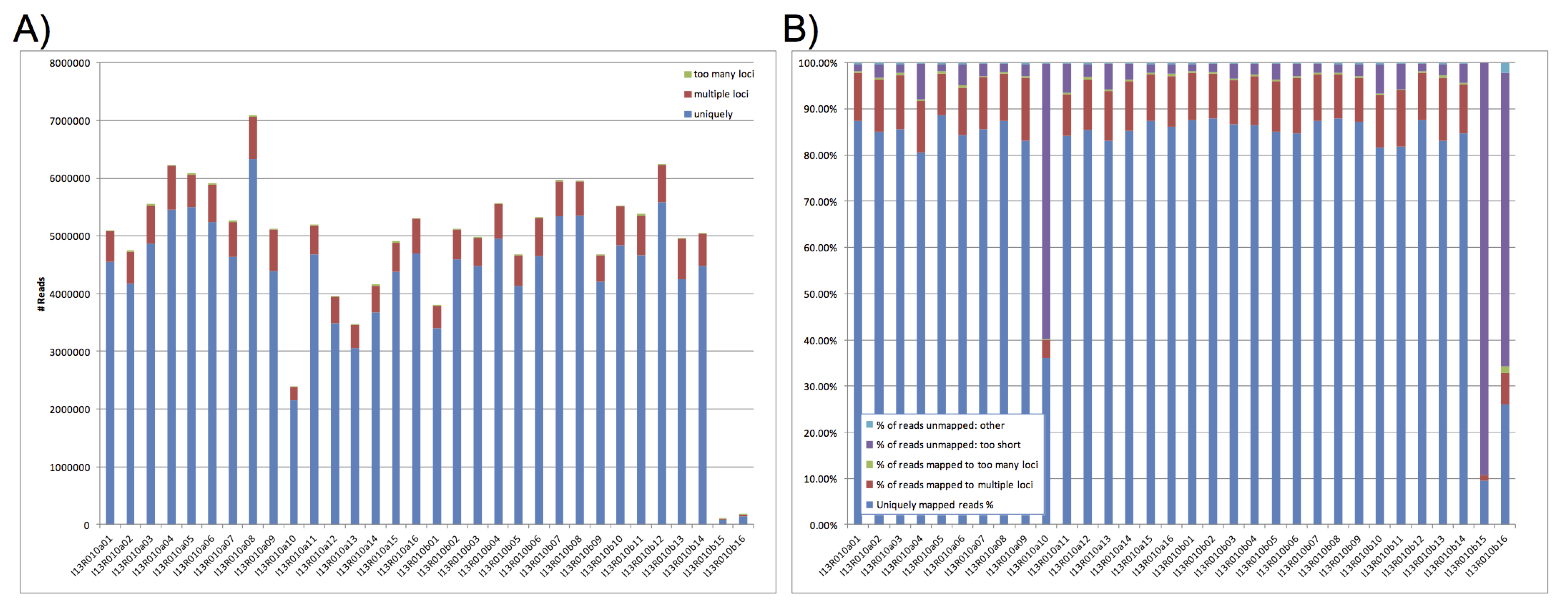

Supplement: S1 Fig — Absolute (A) and relative (B) number of reads mapped to hg19. (TIFF) [file pone.0183526.s001.tiff]

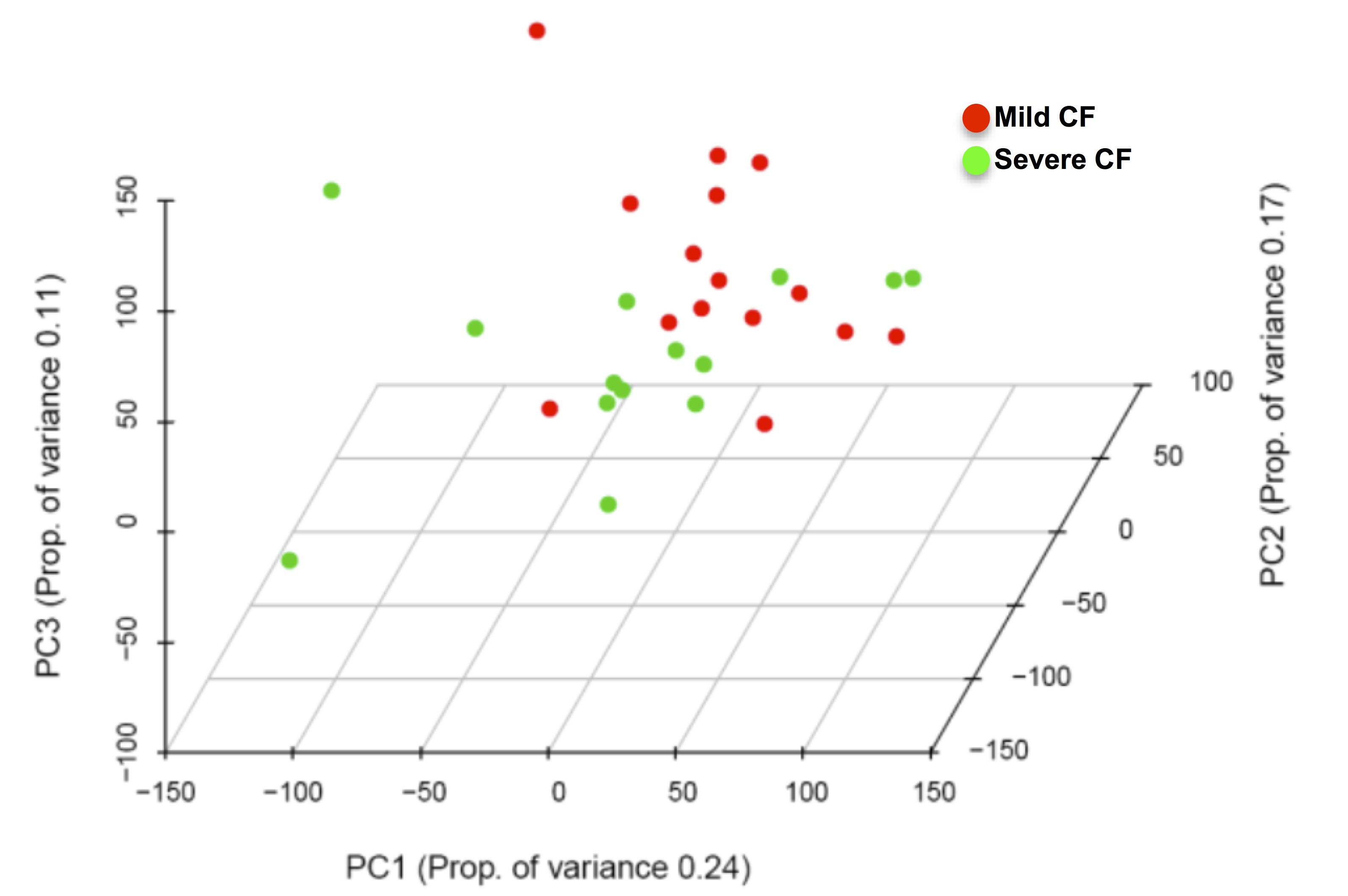

Supplement: S2 Fig — PCA of the filtered CPM counts data to assess the differences in the expression profiles of the two investigated groups. Each point represents one sample; the closer two points are the more similar are the expression profiles. CF manifestation is color-coded. (TIFF) [file pone.0183526.s002.tiff]

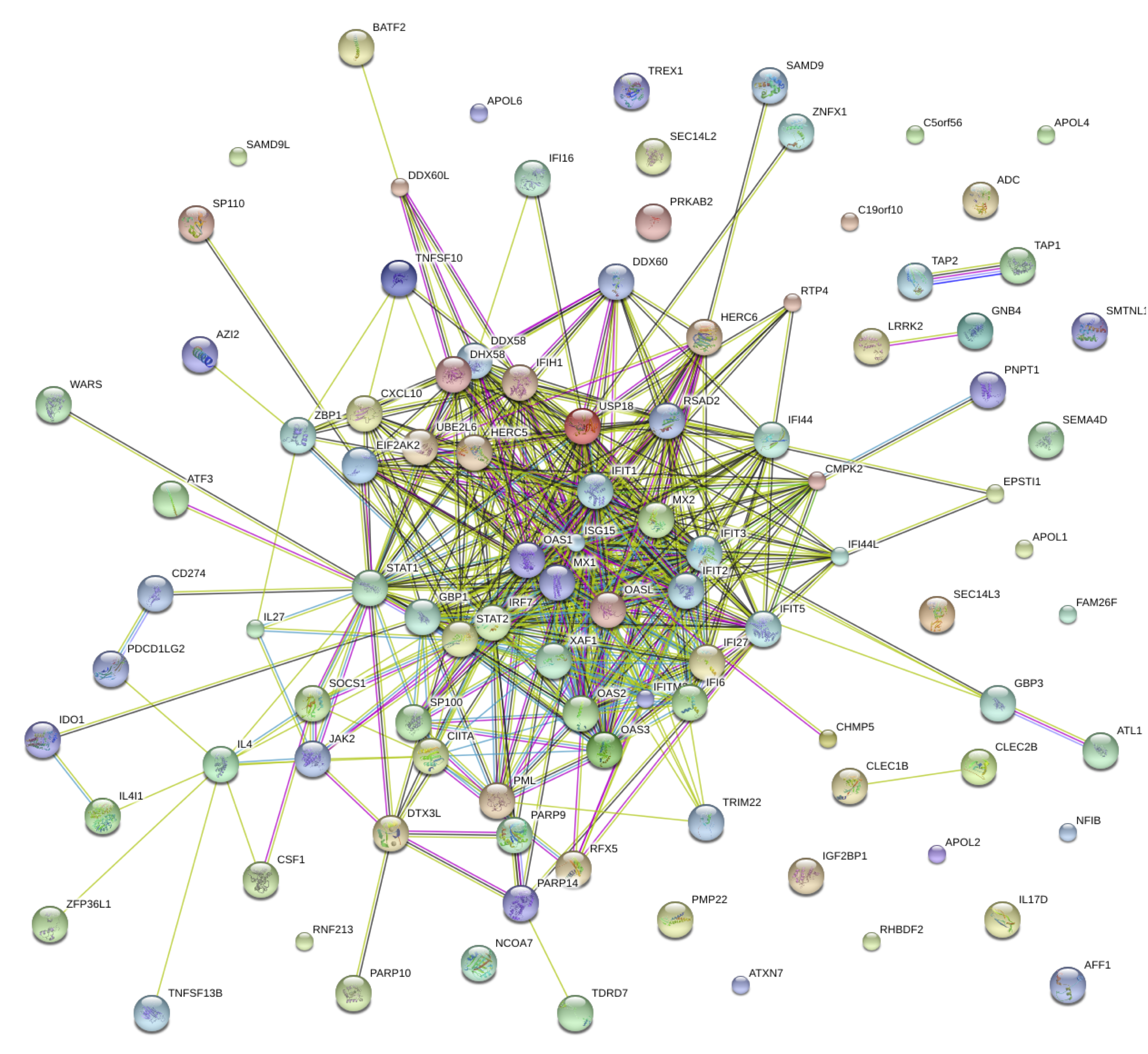

Supplement: S3 Fig — Network of MARA analysis predicts the possible interaction of IRF1,2,8 and STAT2 target genes. (TIFF) [file pone.0183526.s003.tiff]
