# Supplementary material for: Transcriptomic profile of cystic fibrosis patients identifies type I interferon response and ribosomal stalk proteins as potential modifiers of disease severity
Source: PLoS One. 2017 Aug 28;12(8):e0183526. doi: 10.1371/journal.pone.0183526 (PMC5573219; doi:10.1371/journal.pone.0183526)
Supplement: S1 Table — The results of differential expression analysis between mild and severe CF group. The respective expression levels given as a fold change and CPM with corrected P-Values. (PDF) [file pone.0183526.s004.pdf]

Supplementary Table 1

Genes	LogFC	LogCPM	*P* Value	FDR	Group
EGR1	4,75685917	5,23654823	4,97296E-10	3,17722E-06	Over expressed in Mild CF
SFRP1	4,56050125	0,01399744	6,67709E-06	0,00426599	
RSAD2	4,24302736	8,07492536	2,95443E-08	0,000125839	
FOSB	3,64111933	3,92832731	7,47804E-08	0,000191109	
IFIT1	3,46202883	7,97475009	5,77677E-08	0,000184539	
IFI44L	3,4414187	6,91855957	1,77351E-07	0,000241628	
IL8	3,27771097	4,65098567	8,6626E-11	1,10691E-06	
G0S2	3,18564445	4,04214147	1,85408E-05	0,008461214	
OTOF	3,11220905	1,82287055	9,60712E-05	0,024920001	
CXCL10	3,06140183	2,2102772	2,33046E-06	0,001969486	
IFI44	3,0136557	6,57421713	1,60928E-07	0,000241628	
ISG15	2,87741886	6,96415024	4,89784E-06	0,003293926	
CMPK2	2,73073419	5,9613396	1,85287E-06	0,001691141	
EGR3	2,65348939	2,135929	7,71508E-05	0,021907392	
LAMP3	2,6530355	3,09078531	8,09393E-05	0,022483539	
HERC5	2,62284335	6,91854226	2,4661E-06	0,001969486	
IFIT3	2,61640412	8,83331063	9,90497E-07	0,001054714	
OAS3	2,47121912	8,11048018	1,26507E-05	0,00646604	
SIGLEC1	2,4661411	5,05708989	3,02927E-05	0,012486459	
EGR2	2,4117919	1,17992739	0,000244112	0,040389143	
SMTNL1	2,36581672	2,42278185	0,000248532	0,040389143	
USP18	2,30775931	2,2778699	0,000107015	0,025800772	
ABO	2,21426041	0,2243331	0,000267484	0,042196447	
IFIT2	2,19282706	9,3285642	1,37918E-06	0,001355631	
IFI6	2,17676155	7,11215417	2,22774E-05	0,009488674	
SEPT4	2,12672281	2,41362464	4,97384E-05	0,017654364	
FOS	2,11147897	8,34845726	4,04103E-07	0,000469421	
MX1	1,9826741	8,34885163	6,24467E-05	0,0199486	
ABCA13	1,95588032	1,38590224	0,000324446	0,048206653	
PTGS2	1,85933601	6,3563357	9,49467E-08	0,000202205	
OASL	1,71121639	6,62487045	0,000319695	0,048059545	
TPST1	1,64027026	3,83869717	4,07609E-05	0,015318915	
IFIT5	1,60137965	6,37263397	1,44456E-05	0,007099476	
RTP4	1,49459643	3,96403254	0,000191988	0,035282226	
CLEC9A	1,40548726	1,54654361	6,01134E-05	0,0199486	
ZNF684	1,36099526	2,2156351	4,03996E-05	0,015318915	
ZNF117	1,33292188	6,09348048	1,03182E-05	0,005493602	
RPL34	1,30503652	5,34525252	0,000334494	0,049128323	
MS4A3	1,29447171	3,23110638	2,00011E-05	0,008812894	
KY	1,2894184	2,31204488	1,89097E-07	0,000241628	
ZBP1	1,26130826	6,12112739	5,66385E-05	0,019560187	
CD69	1,23796122	3,53302714	7,08259E-06	0,004309587	
SAMD9	1,22328594	7,64574166	0,000232886	0,039258933	
DUSP1	1,20786257	8,83410518	0,000159095	0,032268471	
CHMP5	1,16958162	5,58240199	0,000116703	0,027113313	
RPL31	1,1579116	6,23542853	0,000233501	0,039258933	

Genes	LogFC	LogCPM	*P* Value	FDR	Group
FAM26F	1,10947521	4,18884117	0,00011951	0,027127414	Over expressed in Mild CF
CETN3	1,10115548	1,81181475	0,000314653	0,047864734	
RPS24	1,08438978	6,2394819	0,000285286	0,044455846	
CLEC2B	1,07854663	5,59553273	0,000135647	0,028888203	
POLR2K	1,03727835	3,47452811	0,000122313	0,027127414	
PMAIP1	1,00954679	3,6666252	2,63467E-06	0,001980341	
TMEM123	0,93000559	8,94088988	0,000123133	0,027127414	
B2M	0,89334493	13,2121243	0,000342112	0,049676182	
SPAG1	0,86941069	3,13717339	8,42148E-05	0,02283827	
MRPL47	0,86526561	3,30066822	0,00021638	0,038401404	
LOC338758	0,85338339	2,15060865	0,000195152	0,035282226	
ARRDC3	0,85271369	8,16823747	1,8379E-05	0,008461214	
DDIT3	0,8164075	4,23864419	4,28511E-05	0,015644306	
TPRKB	0,80427373	2,7553625	0,000192612	0,035282226	
HCG27	0,7843849	6,51997721	3,40117E-05	0,013581308	
ZNF92	0,76976199	3,9322455	0,000182428	0,035282226	
NDUFA5	0,76933422	3,73290386	0,000178876	0,035282226	
SCLT1	0,76648461	4,99439004	9,92168E-05	0,024920001	
SASS6	0,76501924	2,74584052	6,16935E-05	0,0199486	
ABHD3	0,76321014	6,47161195	0,00014531	0,029947853	
ZNF708	0,76077654	3,82218108	0,00014056	0,029443841	
FGL2	0,73232659	10,0327795	0,000223373	0,039099522	
ATP6V1G1	0,73071843	6,60372699	7,6332E-05	0,021907392	
SBDSP1	0,70752957	3,18967094	0,000129578	0,028063456	
MBIP	0,66055476	3,13297421	0,000101412	0,024920001	
MALAT1	0,64068412	9,17379658	9,96884E-05	0,024920001	
ZNF136	0,54956276	3,82950058	0,000193051	0,035282226	
COA1	0,54062677	4,75695263	0,000314005	0,047864734	
CRTC3	-0,46378978	6,2799412	7,57566E-05	0,021907392	Over expressed in severe CF
PLD2	-0,52832926	4,51268889	0,000179533	0,035282226	
POU2F2	-0,56166486	7,05797144	0,00023262	0,039258933	
ADORA2A	-0,56418647	5,81254057	6,76041E-05	0,021069404	
SSBP3	-0,56746822	4,76010263	0,000196043	0,035282226	
CDK5RAP2	-0,7402793	6,01407434	7,37192E-05	0,021907392	
AEBP1	-1,0241242	3,52520657	0,000113444	0,026844276	
PTGFRN	-1,12029499	1,44657919	8,5791E-05	0,02283827	
HSD3B7	-1,17987244	2,5789736	0,000249706	0,040389143	
ZNF683	-1,52672894	3,52591536	1,45732E-07	0,000241628	
C4BPA	-3,02639426	3,4706908	3,17421E-06	0,002253333	
AP3B2	-3,15160686	1,69603618	9,72666E-06	0,005403796	
LOC644172	-4,01662914	0,55410415	8,16245E-06	0,004740899	
EPB41L4B	-4,50678292	0,52592381	0,000260297	0,041575945	

Log FC Log of Fold Cange
Log CPM Log of Count Per Million
FDR False Discovery Rate
