# Supplementary material for: Transcriptomic profile of cystic fibrosis patients identifies type I interferon response and ribosomal stalk proteins as potential modifiers of disease severity
Source: PLoS One. 2017 Aug 28;12(8):e0183526. doi: 10.1371/journal.pone.0183526 (PMC5573219; doi:10.1371/journal.pone.0183526)
Supplement: S2 Table — The results of GO enrichement analysis for DEGs that are upregulated in mild and severe CF. Values of fold enrichment of specififed pathways with with corrected P-values were stated. (PDF) [file pone.0183526.s005.pdf]

Supplementary Table 2

GO ID	Term	Total No. Genes	Enriched Genes	% (Fold Enrichment)	*P*-value
GO:0060337	Type I interferon Response	63	10	38.71	1.45E-09
GO:0051607	defense response to virus	169	13	19.76	2.56E-09
GO:0002252	immune effector process	532	16	7.33	4.42E-06
GO:0006950	response to stress	761	17	5.45	9.99E-05
GO:0045047	Protein targetting to ER	90	4	5.30	1.89E-05
GO:0043207	response to external biotic stimulus	876	19	5.29	1.93E-05
GO:0071345	cellular response to cytokine stimulus	652	14	5.24	3.65E-03
GO:0006952	defense response	1262	25	4.83	2.11E-07
GO:0006955	immune response	1169	20	4.17	3.58E-04
GO:0009605	response to external stimulus	1911	26	3.32	2.24E-04
GO:0002376	immune system process	2106	28	3.24	8.85E-05
