# Supplementary material for: Transcriptomic profile of cystic fibrosis patients identifies type I interferon response and ribosomal stalk proteins as potential modifiers of disease severity
Source: PLoS One. 2017 Aug 28;12(8):e0183526. doi: 10.1371/journal.pone.0183526 (PMC5573219; doi:10.1371/journal.pone.0183526)
Supplement: S1 File — The complete raw data of MARA analysis that predicted the IRF1,2,8 and STAT2 activity in RNA-seq. (PDF) [file pone.0183526.s006.pdf]

ISMARA

Project

Final-1

Navigation

All motifs sorted by activity significance

All samples sorted alphabetically

Mean activities

Downloads

Activities table

Activity deltas table

Regulatory interactions

Motifs sorted by significance (text)

Download the whole report

Motif ID: IRF2_STAT2_IRF8_IRF1

Z-value: 5.971

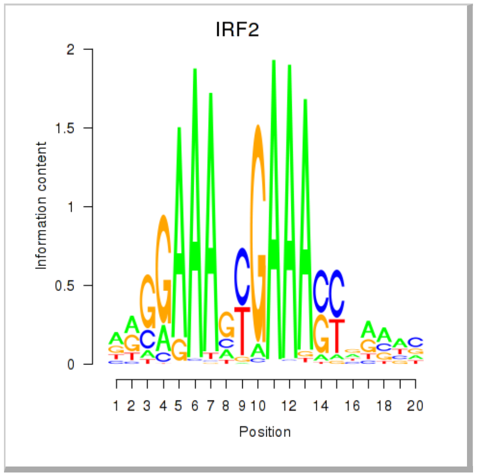

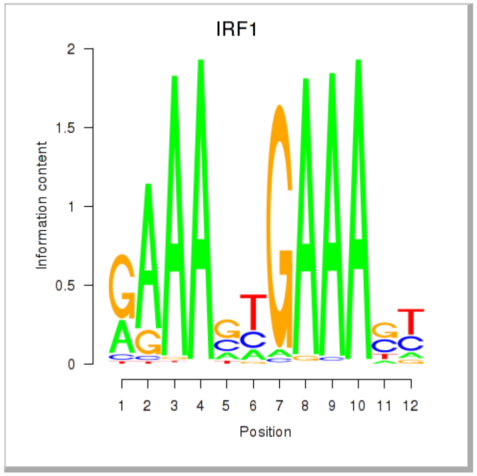

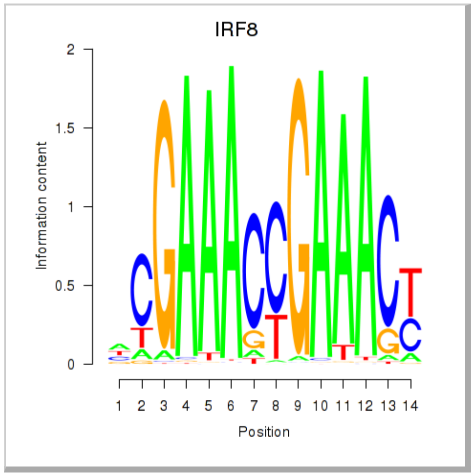

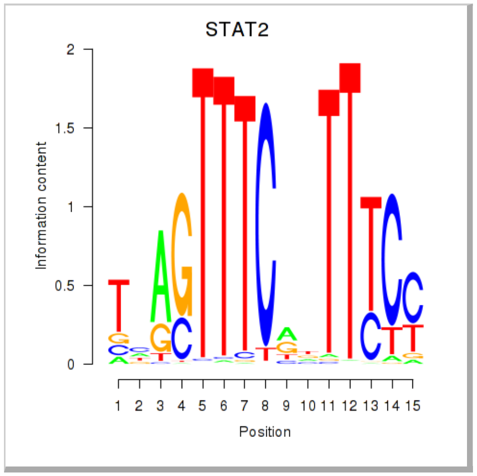

Activity graph

Z-values graph

Top targets

GO analysis

Back to top

Transcription factors associated with IRF2_STAT2_IRF8_IRF1:

Gene Symbol	Entrez ID	Gene Name
IRF1	ENSG00000125347.9	IRF1

ISMARA

Project

Final-1

Navigation

All motifs sorted by activity significance

All samples sorted alphabetically

Mean activities

Downloads

Activities table

Activity deltas table

Regulatory interactions

Motifs sorted by significance (text)

Download the whole report

IRF2	ENSG00000168310.6	IRF2
IRF8	ENSG00000140968.6	IRF8
STAT2	ENSG00000170581.9	STAT2

Activity-expression correlation:

Gene Symbol	Promoter	Pearson corr. coef.	P-value	Plot
STAT2	hg19_v2_chr12_-_56753858_56753930	0.82	2.1e-08	Click!
IRF1	hg19_v2_chr5_-_131826457_131826514	0.70	1.9e-05	Click!
IRF2	hg19_v2_chr4_-_185395672_185395734	0.62	2.8e-04	Click!
IRF8	hg19_v2_chr16_+_85936295_85936444	0.29	1.3e-01	Click!

Activity profile for motif IRF2_STAT2_IRF8_IRF1.

Activity graph

Z-values graph

Top targets

GO analysis

Back to top

ISMARA

Project

Final-1

Navigation

All motifs sorted by activity significance

All samples sorted alphabetically

Mean activities

Downloads

Activities table

Activity deltas table

Regulatory interactions

Motifs sorted by significance (text)

Download the whole report

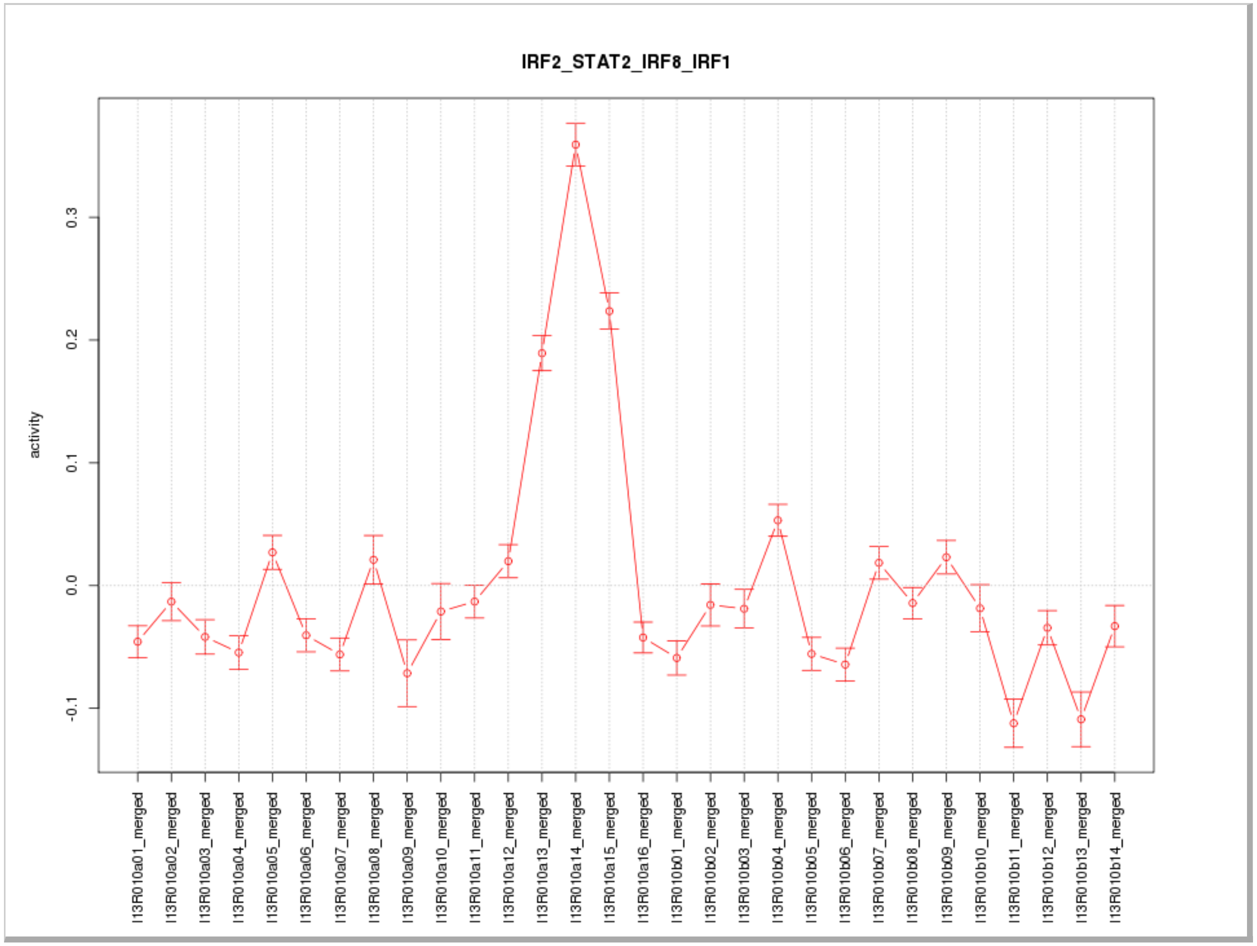

Sorted Z-values histogram for motif IRF2_STAT2_IRF8_IRF1

Activity graph

Z-values graph

Top targets

GO analysis

Back to top

ISMARA

Project

Final-1

Navigation

All motifs sorted by activity significance

All samples sorted alphabetically

Mean activities

Downloads

Activities table

Activity deltas table

Regulatory interactions

Motifs sorted by significance (text)

Download the whole report

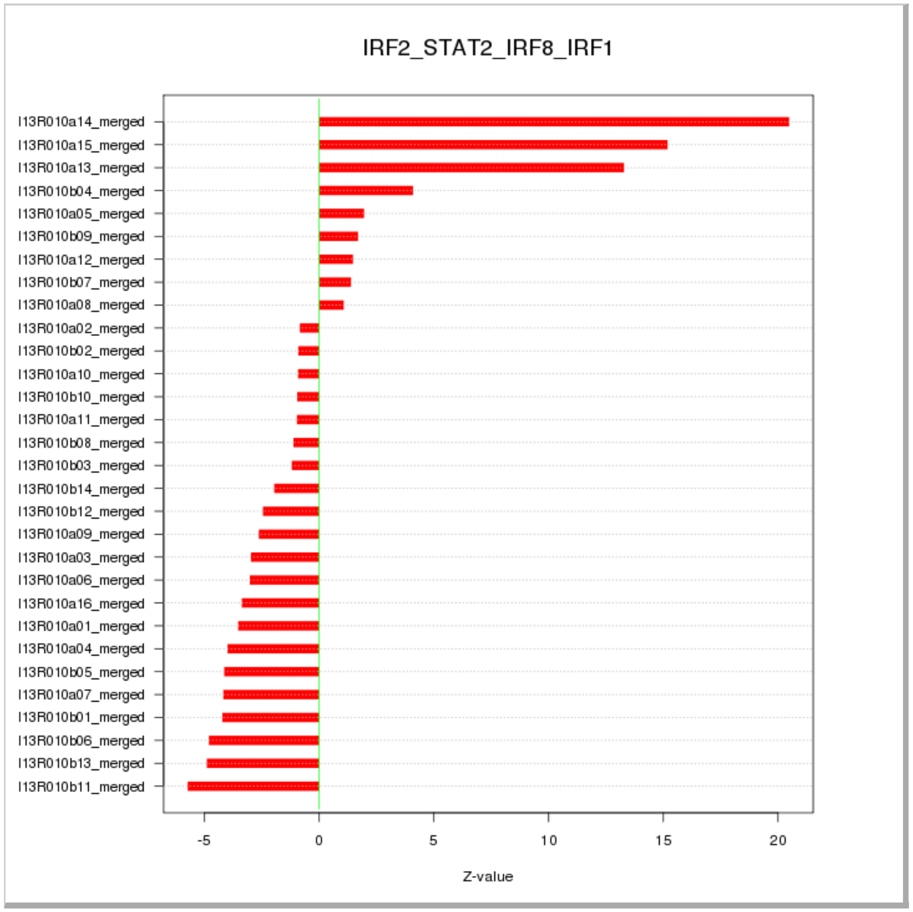

Network of associatons between targets according to the STRING database.

Activity graph

Z-values graph

Top targets

GO analysis

Back to top

ISMARA

Project

Final-1

Navigation

All motifs sorted by activity significance

All samples sorted alphabetically

Mean activities

Downloads

Activities table

Activity deltas table

Regulatory interactions

Motifs sorted by significance (text)

Download the whole report

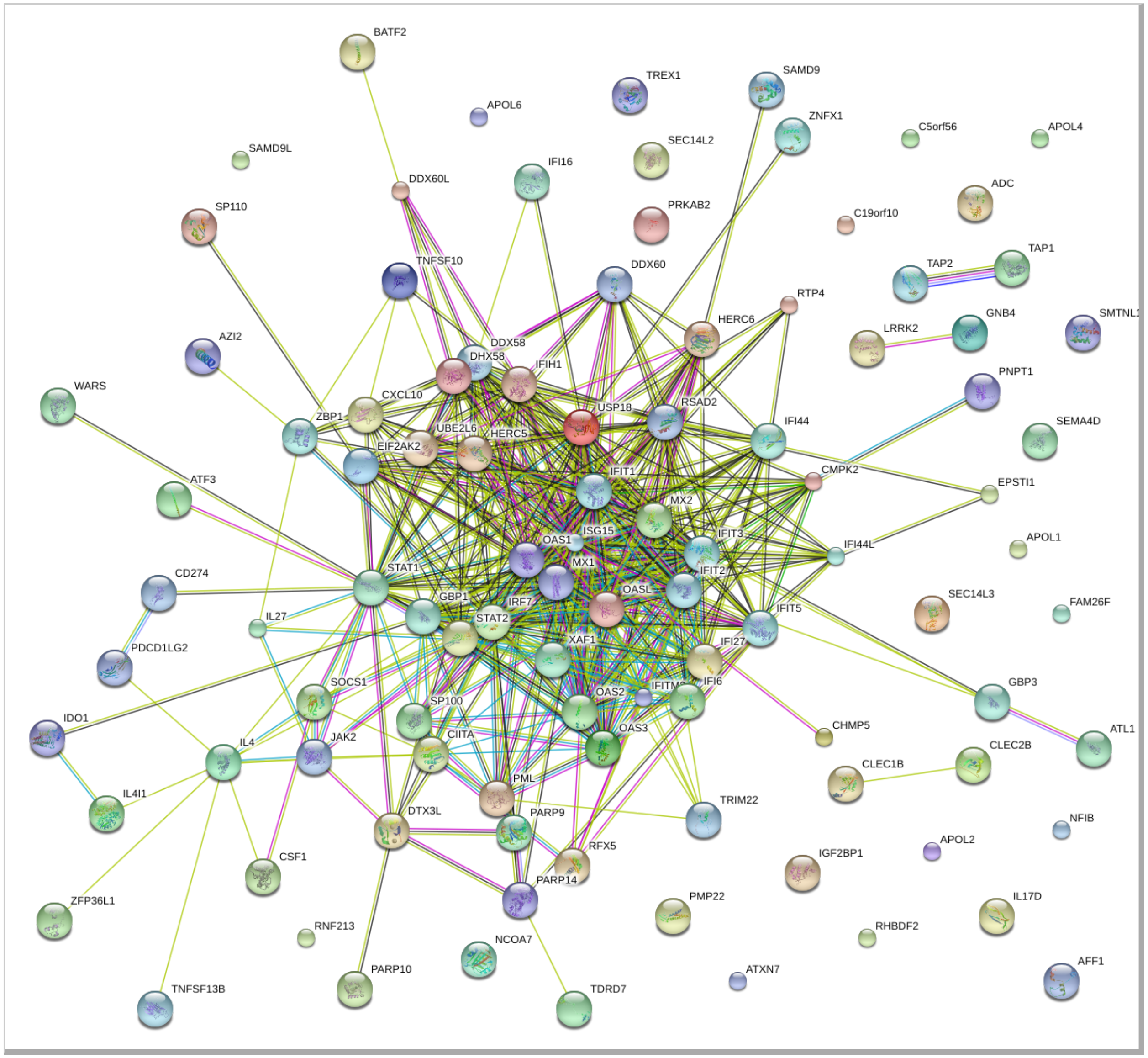

Activity graph

Z-values graph

Top targets

GO analysis

Back to top

First level regulatory network of IRF2_STAT2_IRF8_IRF1

ISMARA

Project

Final-1

Navigation

All motifs sorted by activity significance
All samples sorted alphabetically
Mean activities

Downloads

Activities table
Activity deltas table
Regulatory interactions
Motifs sorted by significance (text)
Download the whole report

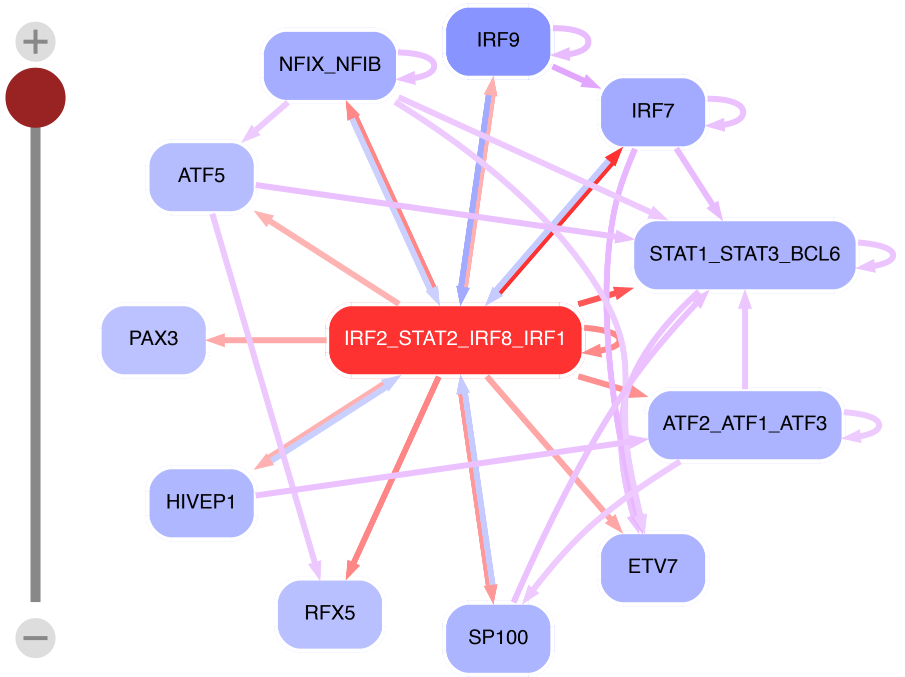

Score threshold: 0.001
Use slider or -/+ buttons to hide/show edges.

View svg image
View png image

Top targets:

Search:
Show All entries
Showing 1 to 200 of 200 entries

Promoter	Score	Refseq	Gene Symbol	Gene Name
chr2_+_7005959	69.597	ENST00000442639.1	RSAD2	radical S-adenosyl methionine domain containing 2
chr2_+_7017796	56.421	ENST00000382040.3	RSAD2	radical S-adenosyl methionine domain containing 2
				interferon-induced protein 4

Activity graph
Z-values graph
Top targets
GO analysis
Back to top

ISMARA

Project

Final-1

Navigation

All motifs sorted by activity significance

All samples sorted alphabetically

Mean activities

Downloads

Activities table

Activity deltas table

Regulatory interactions

Motifs sorted by significance (text)

Download the whole report

Activity graph

Z-values graph

Top targets

GO analysis

Back to top

chr1_+_79086088	56.379	ENST00000370751.5<br>ENST00000342282.3	IFI44L<br>IFI44L	4-like<br>interferon-induced protein 4 4-like
chr10_+_91152303	44.091	ENST00000371804.3	IFIT1	interferon-induced protein with tetratricopeptide repeats 1
chr1_+_948803	40.058	ENST00000379389.4	ISG15	ISG15 ubiquitin-like modifier
chr10_+_91087651	38.585	ENST00000371818.4	IFIT3	interferon-induced protein with tetratricopeptide repeats 3
chr2_-_7005785	38.074	ENST00000458098.1<br>ENST00000256722.5<br>ENST00000404168.1	CMPK2<br>CMPK2	cytidine monophosphate (UMP-CMP) kinase 2, mitochondrial<br>cytidine monophosphate (UMP-CMP) kinase 2, mitochondrial
chr10_+_91092241	37.841	ENST00000371811.4	IFIT3	interferon-induced protein with tetratricopeptide repeats 3
chr12_+_113376157	32.420	ENST00000228928.7	OAS3	2'-5'-oligoadenylate synthetase 3, 100kDa
chr17_+_6659354	31.302	ENST00000574907.1	XAF1	XIAP associated factor 1
chr22_+_18632666	30.767	ENST00000215794.7	USP18	ubiquitin specific peptidase 18
chr4_+_89378261	26.528	ENST00000264350.3	HERC5	HECT and RLD domain containing E3 ubiquitin protein ligase 5
chr17_+_6658878	26.450	ENST00000574394.1	XAF1	XIAP associated factor 1
chr1_+_79115503	25.933	ENST00000370747.4<br>ENST00000438486.1<br>ENST00000545124.1	IFI44<br><br>IFI44	interferon-induced protein 44<br><br>interferon-induced protein 44
chr12_+_113416340	25.685	ENST00000552756.1	OAS2	2'-5'-oligoadenylate synthet

ISMARA

Project

Final-1

Navigation

All motifs sorted by activity significance

All samples sorted alphabetically

Mean activities

Downloads

Activities table

Activity deltas table

Regulatory interactions

Motifs sorted by significance (text)

Download the whole report

Activity graph

Z-values graph

Top targets

GO analysis

Back to top

				ase 2, 69/71kDa
chr17_-_40264321	25.529	ENST00000430773.1 ENST00000413196.2	DHX58 DHX58	DEXH (Asp-Glu-X-His) box polypeptide 58 DEXH (Asp-Glu-X-His) box polypeptide 58
chr12_+_113376249	25.524	ENST00000551007.1 ENST00000548514.1	OAS3 OAS3	2'-5'-oligoadenylate synthetase 3, 100kDa 2'-5'-oligoadenylate synthetase 3, 100kDa
chr4_-_169239921	24.929	ENST00000514995.1 ENST00000393743.3	DDX60 DDX60	DEAD (Asp-Glu-Ala-Asp) box polypeptide 60 DEAD (Asp-Glu-Ala-Asp) box polypeptide 60
chr13_-_43566301	24.729	ENST00000313624.7 ENST00000398762.3 ENST00000313640.7	EPSTI1 EPSTI1	epithelial stromal interaction 1 (breast) epithelial stromal interaction 1 (breast)
chr11_-_64764435	24.560	ENST00000301887.4 ENST00000534177.1	BATF2 BATF2	basic leucine zipper transcription factor, ATF-like 2 basic leucine zipper transcription factor, ATF-like 2
chr14_+_94577074	24.433	ENST00000555744.1 ENST00000444961.1 ENST00000448882.1 ENST00000557098.1 ENST00000554800.1 ENST00000556544.1 ENST00000298902.5 ENST00000555819.1 ENST00000557634.1	IFI27 IFI27	interferon, alpha-inducible protein 27 interferon, alpha-inducible protein 27
chr10_+_91174486	22.921	ENST00000416601.1	IFIT5	interferon-induced protein with tetratricopeptide repeats 5
chr4_+_89300158	22.724	ENST00000502870.1	HERC6	HECT and RLD domain containing E3 ubiquitin protei

				n ligase family member 6
chr4_-_76944621	22.397	ENST00000306602.1	CXCL10	chemokine (C-X-C motif) ligand 10
chr12_+_113416265	21.771	ENST00000449768.2	OAS2	2'-5'-oligoadenylate synthetase 2, 69/71kDa
chr12_+_113416191	21.351	ENST00000342315.4<br>ENST00000392583.2	OAS2<br>OAS2	2'-5'-oligoadenylate synthetase 2, 69/71kDa<br>2'-5'-oligoadenylate synthetase 2, 69/71kDa
chr9_+_5450503	19.893	ENST00000381577.3<br>ENST00000381573.4	CD274<br>CD274	CD274 molecule<br>CD274 molecule
chr21_+_42792442	19.836	ENST00000398600.2	MX1	myxovirus (influenza virus) resistance 1, interferon-inducible protein p78 (mouse)
chr10_+_91061712	19.794	ENST00000371826.3	IFIT2	interferon-induced protein with tetratricopeptide repeats 2
chr3_+_122399697	19.502	ENST00000494811.1	PARP14	poly (ADP-ribose) polymerase family, member 14
chr10_+_91174314	19.292	ENST00000371795.4	IFIT5	interferon-induced protein with tetratricopeptide repeats 5
chr1_-_27998689	17.923	ENST00000339145.4<br>ENST00000362020.4<br>ENST00000361157.6	IFI6<br><br>IFI6	interferon, alpha-inducible protein 6<br><br>interferon, alpha-inducible protein 6
chr17_+_6659153	17.782	ENST00000361842.3<br>ENST00000441631.1<br>ENST00000438512.1<br>ENST00000346752.4	XAF1<br>XAF1	XIAP associated factor 1<br>XIAP associated factor 1
chr3_+_187086120	17.094	ENST00000259030.2	RTP4	receptor (chemosensory) transporter protein 4
chr16_-_28518153	16.848	ENST00000356897.1	IL27	interleukin 27
chr9_+_5510558	16.841	ENST00000397747.3	PDCD1LG2	programmed cell death 1 ligand 2

ISMARA

Project

Final-1

Navigation

All motifs sorted by activity significance

All samples sorted alphabetically

Mean activities

Downloads

Activities table

Activity deltas table

Regulatory interactions

Motifs sorted by significance (text)

Download the whole report

Activity graph

Z-values graph

Top targets

GO analysis

Back to top

chr9_+_5510492	15.654	ENST00000397745.2	PDCD1LG2	programmed cell death 1 ligand 2
chr17_-_40264692	15.241	ENST00000591220.1<br>ENST00000251642.3	DHX58<br>DHX58	DEXH (Asp-Glu-X-His) box polypeptide 58<br>DEXH (Asp-Glu-X-His) box polypeptide 58
chr7_-_92777606	14.970	ENST00000318238.4<br>ENST00000437805.1<br>ENST00000446959.1<br>ENST00000439952.1<br>ENST00000414791.1<br>ENST00000446033.1<br>ENST00000411955.1	SAMD9L<br>SAMD9L	sterile alpha motif domain containing 9-like<br>sterile alpha motif domain containing 9-like
chr11_-_615942	14.786	ENST00000397574.2<br>ENST00000397562.3<br>ENST00000330243.5<br>ENST00000397570.1	IRF7<br>IRF7	interferon regulatory factor 7<br>interferon regulatory factor 7
chr1_-_89488510	14.490	ENST00000564665.1<br>ENST00000370481.4	GBP3<br>GBP3	guanylate binding protein 3<br>guanylate binding protein 3
chr20_-_56195449	14.359	ENST00000541799.1	ZBP1	Z-DNA binding protein 1
chr9_-_32526184	13.721	ENST00000545044.1<br>ENST00000379868.1	DDX58<br>DDX58	DEAD (Asp-Glu-Ala-Asp) box polypeptide 58<br>DEAD (Asp-Glu-Ala-Asp) box polypeptide 58
chr1_-_89531041	13.642	ENST00000370473.4	GBP1	guanylate binding protein 1, interferon-inducible
chr9_-_32526299	12.917	ENST00000379883.2<br>ENST00000379882.1	DDX58<br>DDX58	DEAD (Asp-Glu-Ala-Asp) box polypeptide 58<br>DEAD (Asp-Glu-Ala-Asp) box polypeptide 58
chr2_-_163175133	12.832	ENST00000263642.2<br>ENST00000421365.2	IFIH1<br>IFIH1	interferon induced with helicase C domain 1

ISMARA

Project

Final-1

Navigation

All motifs sorted by activity significance

All samples sorted alphabetically

Mean activities

Downloads

Activities table

Activity deltas table

Regulatory interactions

Motifs sorted by significance (text)

Download the whole report

				interferon induced with helicase C domain 1
chr12_+_113344811	12.371	ENST00000551241.1<br>ENST00000553185.1<br>ENST00000550689.1	OAS1<br>OAS1	2'-5'-oligoadenylate synthetase 1, 40/46kDa<br>2'-5'-oligoadenylate synthetase 1, 40/46kDa
chr2_-_37384175	12.008	ENST00000390013.3<br>ENST00000411537.2<br>ENST00000233057.4<br>ENST00000395127.2	EIF2AK2<br>EIF2AK2	eukaryotic translation initiation factor 2-alpha kinase 2<br>eukaryotic translation initiation factor 2-alpha kinase 2
chr11_-_615570	12.006	ENST00000525445.1<br>ENST00000348655.6<br>ENST00000397566.1	IRF7<br>IRF7	interferon regulatory factor 7<br>interferon regulatory factor 7
chr3_+_122283175	11.651	ENST00000383661.3	DTX3L	deltex 3-like (Drosophila)
chr20_-_56195525	11.602	ENST00000371173.3<br>ENST00000395822.3<br>ENST00000340462.4<br>ENST00000343535.4	ZBP1<br>ZBP1	Z-DNA binding protein 1<br>Z-DNA binding protein 1
chr11_+_5711010	11.426	ENST00000454828.1	TRIM22	tripartite motif containing 22
chr5_+_131746575	11.406	ENST00000337752.2<br>ENST00000378947.3<br>ENST00000407797.1	C5orf56<br>C5orf56	chromosome 5 open reading frame 56<br>chromosome 5 open reading frame 56
chr2_-_191878681	11.226	ENST00000409465.1	STAT1	signal transducer and activator of transcription 1, 91kDa
chr9_+_100174232	11.106	ENST00000355295.4	TDRD7	tudor domain containing 7
chr14_-_100841794	10.771	ENST00000556295.1<br>ENST00000554820.1	WARS<br>WARS	tryptophanyl-tRNA synthetase<br>tryptophanyl-tRNA synthetase

Activity graph

Z-values graph

Top targets

GO analysis

Back to top

ISMARA

Project

Final-1

Navigation

All motifs sorted by activity significance
All samples sorted alphabetically
Mean activities

Downloads

Activities table
Activity deltas table
Regulatory interactions
Motifs sorted by significance (text)
Download the whole report

Activity graph
Z-values graph
Top targets
GO analysis
Back to top

chr4_-_169401628	10.759	ENST00000514748.1<br>ENST00000512371.1<br>ENST00000260184.7<br>ENST00000505890.1<br>ENST00000511577.1	DDX60L<br>DDX60L	DEAD (Asp-Glu-Ala-Asp) box polypeptide 60-like<br>DEAD (Asp-Glu-Ala-Asp) box polypeptide 60-like
chr3_+_122399444	10.698	ENST00000474629.2	PARP14	poly (ADP-ribose) polymerase family, member 14
chr9_+_100174344	10.510	ENST00000422139.2	TDRD7	tudor domain containing 7
chr3_-_122283079	10.248	ENST00000471785.1<br>ENST00000466126.1	PARP9<br>PARP9	poly (ADP-ribose) polymerase family, member 9<br>poly (ADP-ribose) polymerase family, member 9
chr21_+_42797958	10.197	ENST00000419044.1	MX1	myxovirus (influenza virus) resistance 1, interferon-inducible protein p78 (mouse)
chr21_+_42798158	10.118	ENST00000441677.1	MX1	myxovirus (influenza virus) resistance 1, interferon-inducible protein p78 (mouse)
chr21_+_42798124	10.063	ENST00000417963.1	MX1	myxovirus (influenza virus) resistance 1, interferon-inducible protein p78 (mouse)
chr11_-_57334732	9.683	ENST00000527022.1<br>ENST00000526659.1	UBE2L6<br>UBE2L6	ubiquitin-conjugating enzyme E2L 6<br>ubiquitin-conjugating enzyme E2L 6
chr2_-_191878874	9.548	ENST00000392322.3<br>ENST00000392323.2<br>ENST00000424722.1<br>ENST00000361099.3	STAT1<br>STAT1	signal transducer and activator of transcription 1, 91kDa<br>signal transducer and activator of transcription 1, 91kDa
chr17_+_78234625	9.508	ENST00000508628.2<br>ENST00000582970.1	RNF213	ring finger protein 213

ISMARA

Project

Final-1

Navigation

All motifs sorted by activity significance

All samples sorted alphabetically

Mean activities

Downloads

Activities table

Activity deltas table

Regulatory interactions

Motifs sorted by significance (text)

Download the whole report

		ENST00000456466.1<br>ENST00000319921.4	RNF213	ring finger protein 213
chr3_-_122283424	9.493	ENST00000360356.2<br>ENST00000477522.2	PARP9<br>PARP9	poly (ADP-ribose) polymerase family, member 9<br>poly (ADP-ribose) polymerase family, member 9
chr11_+_5710919	9.441	ENST00000425490.1<br>ENST00000379965.3	TRIM22<br>TRIM22	tripartite motif containing 22<br>tripartite motif containing 22
chr22_+_36649170	9.417	ENST00000440669.2<br>ENST00000438034.1<br>ENST00000427990.1<br>ENST00000347595.7<br>ENST00000397279.4<br>ENST00000433768.1	APOL1<br>APOL1	apolipoprotein L, 1<br>apolipoprotein L, 1
chr3_+_63897605	8.963	ENST00000487717.1	ATXN7	ataxin 7
chr22_-_36635598	8.802	ENST00000454728.1	APOL2	apolipoprotein L, 2
chr3_-_179169330	8.471	ENST00000232564.3	GNB4	guanine nucleotide binding protein (G protein), beta polypeptide 4
chr22_-_36600727	8.436	ENST00000328429.4<br>ENST00000397275.2	APOL4<br>APOL4	apolipoprotein L, 4<br>apolipoprotein L, 4
chr17_-_74489215	8.428	ENST00000585701.1<br>ENST00000591192.1<br>ENST00000589526.1	RHBDF2<br>RHBDF2	rhomboid 5 homolog 2 (Drosophila)<br>rhomboid 5 homolog 2 (Drosophila)
chr9_+_33264861	8.355	ENST00000223500.8	CHMP5	charged multivesicular body protein 5
chr22_+_36044411	8.329	ENST00000409652.4	APOL6	apolipoprotein L, 6
chr12_+_113344755	8.308	ENST00000550883.1	OAS1	2'-5'-oligoadenylate synthetase 1, 40/46kDa
chr3_-_122283100	8.227	ENST00000462315.1<br>ENST00000492382.1	PARP9<br>PARP9	poly (ADP-ribose) polymerase family, member 9<br>poly (ADP-ribose) polymerase family, member 9
				charged multivesicular bod

Activity graph

Z-values graph

Top targets

GO analysis

Back to top

ISMARA

Project

Final-1

Navigation

All motifs sorted by activity significance

All samples sorted alphabetically

Mean activities

Downloads

Activities table

Activity deltas table

Regulatory interactions

Motifs sorted by significance (text)

Download the whole report

Activity graph

Z-values graph

Top targets

GO analysis

Back to top

chr9_+_33265011	8.208	ENST00000419016.2	CHMP5	y protein 5
chr8_-_145060593	8.144	ENST00000313059.5<br>ENST00000524918.1<br>ENST00000313028.7<br>ENST00000525773.1	PARP10<br><br>PARP10	poly (ADP-ribose) polymerase family, member 10<br><br>poly (ADP-ribose) polymerase family, member 10
chr21_+_42733870	8.122	ENST00000330714.3<br>ENST00000436410.1<br>ENST00000435611.1	MX2<br><br>MX2	myxovirus (influenza virus) resistance 2 (mouse)<br><br>myxovirus (influenza virus) resistance 2 (mouse)
chr21_+_42798094	8.073	ENST00000424365.1<br>ENST00000398598.3<br>ENST00000455164.2	MX1<br>MX1	myxovirus (influenza virus) resistance 1, interferon-inducible protein p78 (mouse)<br>myxovirus (influenza virus) resistance 1, interferon-inducible protein p78 (mouse)
chr6_-_32821599	7.992	ENST00000354258.4	TAP1	transporter 1, ATP-binding cassette, sub-family B (MDR/TAP)
chr2_-_166702601	7.951	ENST00000428888.1	AC009495.4	AC009495.4
chr6_+_37400974	7.883	ENST00000373451.4<br>ENST00000455891.1	CMTR1<br>CMTR1	cap methyltransferase 1<br>cap methyltransferase 1
chr14_-_100841670	7.821	ENST00000555410.1<br>ENST00000557297.1<br>ENST00000555813.1<br>ENST00000557135.1<br>ENST00000556698.1<br>ENST00000554509.1	WARS<br>WARS	tryptophanyl-tRNA synthetase<br>tryptophanyl-tRNA synthetase
chr15_+_74287118	7.658	ENST00000563500.1	PML	promyelocytic leukemia
chr12_+_40618764	7.654	ENST00000343742.2	LRRK2	leucine-rich repeat kinase 2
				family with sequence simila

ISMARA

Project

Final-1

Navigation

All motifs sorted by activity significance

All samples sorted alphabetically

Mean activities

Downloads

Activities table

Activity deltas table

Regulatory interactions

Motifs sorted by significance (text)

Download the whole report

Activity graph

Z-values graph

Top targets

GO analysis

Back to top

chr6_+_116782527	7.551	ENST00000368606.3<br>ENST00000368605.1	FAM26F<br>FAM26F	rity 26, member F<br>family with sequence simila<br>rity 26, member F
chr9_+_4985016	7.498	ENST00000539801.1	JAK2	Janus kinase 2
chr15_+_74287035	7.444	ENST00000359928.4<br>ENST00000395132.2<br>ENST00000268059.6<br>ENST00000354026.6<br>ENST00000268058.3<br>ENST00000565898.1<br>ENST00000569477.1<br>ENST00000569965.1<br>ENST00000567543.1<br>ENST00000436891.3<br>ENST00000435786.2<br>ENST00000564428.1	PML<br>PML	promyelocytic leukemia<br>promyelocytic leukemia
chr1_-_146644036	7.404	ENST00000425272.2	PRKAB2	protein kinase, AMP-activat<br>ed, beta 2 non-catalytic sub<br>unit
chr11_-_57335280	7.139	ENST00000287156.4	UBE2L6	ubiquitin-conjugating enzy<br>me E2L 6
chr19_+_17516624	7.107	ENST00000596322.1<br>ENST00000600008.1<br>ENST00000601885.1	CTD-2521M24.9	CTD-2521M24.9
chr12_+_113344582	7.068	ENST00000452357.2<br>ENST00000202917.5<br>ENST00000445409.2	OAS1<br>OAS1	2'-5'-oligoadenylate synthet<br>ase 1, 40/46kDa<br>2'-5'-oligoadenylate synthet<br>ase 1, 40/46kDa
chr15_+_74287009	6.858	ENST00000395135.3	PML	promyelocytic leukemia
chr13_+_108922228	6.837	ENST00000542136.1	TNFSF13B	tumor necrosis factor (ligan<br>d) superfamily, member 13<br>b
chr1_-_151319654	6.746	ENST00000412774.1<br>ENST00000430227.1	RFX5<br>RFX5	regulatory factor X, 5 (influe<br>nces HLA class II expressio<br>n)<br>regulatory factor X, 5 (influe

ISMARA

Project

Final-1

Navigation

All motifs sorted by activity significance

All samples sorted alphabetically

Mean activities

Downloads

Activities table

Activity deltas table

Regulatory interactions

Motifs sorted by significance (text)

Download the whole report

Activity graph

Z-values graph

Top targets

GO analysis

Back to top

				nces HLA class II expression)
chr16_-_11350036	6.717	ENST00000332029.2	SOCS1	suppressor of cytokine signaling 1
chr9_-_92020841	6.714	ENST00000433650.1	SEMA4D	sema domain, immunoglobulin domain (Ig), transmembrane domain (TM) and short cytoplasmic domain, (semaphorin) 4D
chr22_-_36635225	6.691	ENST00000529194.1	APOL2	apolipoprotein L, 2
chr5_+_132009675	6.681	ENST00000350025.2<br>ENST00000231449.2	IL4<br>IL4	interleukin 4<br>interleukin 4
chr9_-_14322319	6.643	ENST00000606230.1	NFIB	nuclear factor I/B
chr3_-_28389922	6.587	ENST00000415852.1	AZI2	5-azacytidine induced 2
chr3_-_172241250	6.485	ENST00000241261.2<br>ENST00000420541.2	TNFSF10<br>TNFSF10	tumor necrosis factor (ligand) superfamily, member 10<br>tumor necrosis factor (ligand) superfamily, member 10
chr12_-_56753858	6.449	ENST00000314128.4<br>ENST00000557235.1<br>ENST00000418572.2	STAT2<br><br>STAT2	signal transducer and activator of transcription 2, 113kDa<br><br>signal transducer and activator of transcription 2, 113kDa
chr6_+_126240442	6.422	ENST00000444128.1<br>ENST00000448104.1<br>ENST00000438495.1	NCOA7<br>NCOA7	nuclear receptor coactivator 7<br>nuclear receptor coactivator 7
chr2_-_55920952	6.390	ENST00000447944.2	PNPT1	polyribonucleotide nucleotidyltransferase 1
chr3_-_28390415	6.265	ENST00000414162.1<br>ENST00000420543.2	AZI2<br>AZI2	5-azacytidine induced 2<br>5-azacytidine induced 2
chr11_+_57308979	6.187	ENST00000457912.1	SMTNL1	smoothelin-like 1
				transporter 2, ATP-binding

ISMARA

Project

Final-1

Navigation

All motifs sorted by activity significance

All samples sorted alphabetically

Mean activities

Downloads

Activities table

Activity deltas table

Regulatory interactions

Motifs sorted by significance (text)

Download the whole report

chr6_-_32806483	6.166	ENST00000374899.4	TAP2	cassette, sub-family B (MDR/TAP)
chr7_-_92747269	6.074	ENST00000379958.2<br>ENST00000446617.1	SAMD9<br>SAMD9	sterile alpha motif domain containing 9<br>sterile alpha motif domain containing 9
chr22_+_36649056	5.885	ENST00000397278.3<br>ENST00000422706.1<br>ENST00000426053.1<br>ENST00000319136.4	APOL1<br><br><br>APOL1	apolipoprotein L, 1<br><br><br>apolipoprotein L, 1
chr11_-_321050	5.869	ENST00000399808.4	IFITM3	interferon induced transmembrane protein 3
chr1_+_212782012	5.775	ENST00000341491.4<br>ENST00000366985.1	ATF3<br>ATF3	activating transcription factor 3<br>activating transcription factor 3
chr14_-_100841930	5.724	ENST00000553934.1<br>ENST00000555031.1<br>ENST00000553395.1<br>ENST00000553545.1<br>ENST00000344102.5<br>ENST00000556338.1<br>ENST00000392882.2	WARS<br>WARS	tryptophanyl-tRNA synthetase<br>tryptophanyl-tRNA synthetase
chr12_-_121476750	5.708	ENST00000543677.1	OASL	2'-5'-oligoadenylate synthetase-like
chr14_-_69262789	5.658	ENST00000557022.1	ZFP36L1	ZFP36 ring finger protein-like 1
chr12_-_10022735	5.643	ENST00000228438.2	CLEC2B	C-type lectin domain family 2, member B
chr12_-_121476959	5.633	ENST00000339275.5	OASL	2'-5'-oligoadenylate synthetase-like
chr14_-_69262947	5.607	ENST00000557086.1	ZFP36L1	ZFP36 ring finger protein-like 1
				indoleamine 2,3-dioxygena

Activity graph

Z-values graph

Top targets

GO analysis

Back to top

ISMARA

Project

Final-1

Navigation

All motifs sorted by activity significance

All samples sorted alphabetically

Mean activities

Downloads

Activities table

Activity deltas table

Regulatory interactions

Motifs sorted by significance (text)

Download the whole report

chr8_+_39770803	5.606	ENST00000518237.1	IDO1	se 1
chr2_-_231084617	5.601	ENST00000409815.2	SP110	SP110 nuclear body protein
chr3_-_28390298	5.538	ENST00000457172.1	AZI2	5-azacytidine induced 2
chr22_-_36635563	5.496	ENST00000451256.2	APOL2	apolipoprotein L, 2
chr1_+_158979686	5.448	ENST00000368132.3<br>ENST00000295809.7	IFI16<br>IFI16	interferon, gamma-inducible protein 16<br>interferon, gamma-inducible protein 16
chr1_+_158979792	5.367	ENST00000430894.2<br>ENST00000359709.3	IFI16<br>IFI16	interferon, gamma-inducible protein 16<br>interferon, gamma-inducible protein 16
chr12_-_121477039	5.319	ENST00000257570.5	OASL	2'-5'-oligoadenylate synthetase-like
chr13_+_108921977	5.304	ENST00000375887.4<br>ENST00000430559.1	TNFSF13B<br>TNFSF13B	tumor necrosis factor (ligand) superfamily, member 13b<br>tumor necrosis factor (ligand) superfamily, member 13b
chr19_+_17516494	5.298	ENST00000534306.1	CTD-2521M24.9	CTD-2521M24.9
chr1_+_158979680	5.282	ENST00000340979.6<br>ENST00000368131.4	IFI16<br>IFI16	interferon, gamma-inducible protein 16<br>interferon, gamma-inducible protein 16
chr14_-_69262916	5.257	ENST00000553375.1	ZFP36L1	ZFP36 ring finger protein-like 1
chr19_-_50400125	5.245	ENST00000593956.1	IL4I1	interleukin 4 induced 1
chr1_+_110453608	5.239	ENST00000369801.1	CSF1	colony stimulating factor 1 (macrophage)
chr16_+_10972818	5.164	ENST00000576601.1	CIITA	class II, major histocompatibility complex, transactivator
chr20_-_47894936	5.099	ENST00000371754.4	ZNFX1	zinc finger, NFX1-type containing 1

Activity graph

Z-values graph

Top targets

GO analysis

Back to top

ISMARA

Project

Final-1

Navigation

All motifs sorted by activity significance

All samples sorted alphabetically

Mean activities

Downloads

Activities table

Activity deltas table

Regulatory interactions

Motifs sorted by significance (text)

Download the whole report

Activity graph

Z-values graph

Top targets

GO analysis

Back to top

chr3_+_48507210	5.028	ENST00000444177.1<br>ENST00000433541.1<br>ENST00000422277.2<br>ENST00000436480.2	TREX1<br>TREX1	three prime repair exonuclease 1<br>three prime repair exonuclease 1
chr2_+_231280954	4.876	ENST00000341950.4<br>ENST00000409824.1<br>ENST00000409341.1<br>ENST00000409112.1<br>ENST00000340126.4	SP100<br>SP100	SP100 nuclear antigen<br>SP100 nuclear antigen
chr4_+_87928140	4.852	ENST00000307808.6	AFF1	AF4/FMR2 family, member 1
chr1_+_113217073	4.838	ENST00000369645.1	MOV10	Mov10, Moloney leukemia virus 10, homolog (mouse)
chr2_+_231280908	4.828	ENST00000432979.1<br>ENST00000427101.2	SP100<br>SP100	SP100 nuclear antigen<br>SP100 nuclear antigen
chr14_-_67955426	4.828	ENST00000554480.1	TMEM229B	transmembrane protein 229B
chr17_-_54991369	4.825	ENST00000537230.1	TRIM25	tripartite motif containing 25
chr11_-_104916034	4.811	ENST00000375704.3<br>ENST00000528513.1<br>ENST00000375706.2	CARD16<br>CARD16	caspase recruitment domain family, member 16<br>caspase recruitment domain family, member 16
chr1_+_113217345	4.802	ENST00000357443.2	MOV10	Mov10, Moloney leukemia virus 10, homolog (mouse)
chr6_-_32806506	4.799	ENST00000374897.2<br>ENST00000452392.2	TAP2<br>TAP2	transporter 2, ATP-binding cassette, sub-family B (MDR/TAP)<br>Uncharacterized protein
chr1_+_113217043	4.774	ENST00000413052.2	MOV10	Mov10, Moloney leukemia virus 10, homolog (mouse)
chr14_+_35451880	4.773	ENST00000555746.1<br>ENST00000554803.1	SRP54<br>SRP54	signal recognition particle 54kDa

ISMARA

Project

Final-1

Navigation

All motifs sorted by activity significance
All samples sorted alphabetically
Mean activities

Downloads

Activities table
Activity deltas table
Regulatory interactions
Motifs sorted by significance (text)
Download the whole report

Activity graph
Z-values graph
Top targets
GO analysis
Back to top

				signal recognition particle 5 4kDa
chr7_+_18535321	4.741	ENST00000413380.1<br>ENST00000430454.1	HDAC9<br>HDAC9	histone deacetylase 9<br>histone deacetylase 9
chr1_-_150693318	4.665	ENST00000442853.1<br>ENST00000368995.4<br>ENST00000368993.2<br>ENST00000361824.2<br>ENST00000322343.7	HORMAD1<br>HORMAD1	HORMA domain containing 1<br>HORMA domain containing 1
chr2_+_85811525	4.651	ENST00000306384.4	VAMP5	vesicle-associated membrane protein 5
chr15_+_89182156	4.649	ENST00000379224.5	ISG20	interferon stimulated exonuclease gene 20kDa
chr6_+_32821924	4.619	ENST00000374859.2<br>ENST00000453265.2	PSMB9<br>PSMB9	proteasome (prosome, macropain) subunit, beta type, 9<br>proteasome (prosome, macropain) subunit, beta type, 9
chr3_-_146262352	4.556	ENST00000462666.1	PLSCR1	phospholipid scramblase 1
chr6_+_25963020	4.518	ENST00000357085.3	TRIM38	tripartite motif containing 38
chrX_+_37639302	4.490	ENST00000545017.1<br>ENST00000536160.1	CYBB<br>CYBB	cytochrome b-245, beta polypeptide<br>cytochrome b-245, beta polypeptide
chr3_+_122283064	4.466	ENST00000296161.4	DTX3L	deltex 3-like (Drosophila)
chr3_-_146262428	4.422	ENST00000486631.1	PLSCR1	phospholipid scramblase 1
chr19_-_17516449	4.420	ENST00000252593.6	BST2	bone marrow stromal cell antigen 2
chr9_+_26746951	4.411	ENST00000523363.1	RP11-18A15.1	RP11-18A15.1
chr1_+_113217309	4.367	ENST00000369644.1<br>ENST00000544796.1	MOV10<br>MOV10	Mov10, Moloney leukemia virus 10, homolog (mouse)<br>Mov10, Moloney leukemia virus 10, homolog (mouse)

ISMARA

Project

Final-1

Navigation

All motifs sorted by activity significance

All samples sorted alphabetically

Mean activities

Downloads

Activities table

Activity deltas table

Regulatory interactions

Motifs sorted by significance (text)

Download the whole report

Activity graph

Z-values graph

Top targets

GO analysis

Back to top

chr12_-_118498911	4.337	ENST00000544233.1	WSB2	WD repeat and SOCS box containing 2
chr4_+_100737954	4.286	ENST00000296414.7<br>ENST00000512369.1	DAPP1<br>DAPP1	dual adaptor of phosphotyrosine and 3-phosphoinositides<br>dual adaptor of phosphotyrosine and 3-phosphoinositides
chr3_-_28390581	4.271	ENST00000479665.1	AZI2	5-azacytidine induced 2
chr11_-_104905840	4.230	ENST00000436863.3<br>ENST00000526568.1<br>ENST00000393136.4<br>ENST00000531166.1<br>ENST00000534497.1<br>ENST00000527979.1<br>ENST00000446369.1<br>ENST00000353247.5<br>ENST00000528974.1<br>ENST00000533400.1<br>ENST00000525825.1	CASP1<br>CASP1	caspase 1, apoptosis-related cysteine peptidase<br>caspase 1, apoptosis-related cysteine peptidase
chr8_+_27184320	4.218	ENST00000522517.1	PTK2B	protein tyrosine kinase 2 beta
chr2_-_166651152	4.198	ENST00000412248.1<br>ENST00000431484.1	GALNT3<br>GALNT3	UDP-N-acetyl-alpha-D-galactosamine:polypeptide N-acetylgalactosaminyltransferase 3 (GalNAc-T3)<br>UDP-N-acetyl-alpha-D-galactosamine:polypeptide N-acetylgalactosaminyltransferase 3 (GalNAc-T3)
chr11_-_104972158	4.141	ENST00000598974.1<br>ENST00000593315.1<br>ENST00000594519.1<br>ENST00000415981.2	CASP1	caspase 1, apoptosis-related cysteine peptidase<br>caspase recruitment domai

ISMARA

Project

Final-1

Navigation

All motifs sorted by activity significance

All samples sorted alphabetically

Mean activities

Downloads

Activities table

Activity deltas table

Regulatory interactions

Motifs sorted by significance (text)

Download the whole report

Activity graph

Z-values graph

Top targets

GO analysis

Back to top

		ENST00000525374.1<br>ENST00000375707.1	CARD16<br>CARD17	n family, member 16<br>caspase recruitment domain family, member 17
chr8_+_144640499	4.128	ENST00000534018.1<br>ENST00000525721.1	GSDMD<br>GSDMD	gasdermin D<br>gasdermin D
chr3_-_28390120	4.128	ENST00000334100.6	AZI2	5-azacytidine induced 2
chr19_+_17516909	4.108	ENST00000600514.1<br>ENST00000601007.1<br>ENST00000594913.1<br>ENST00000599975.1	MVB12A<br>CTD-2521M24.9	multivesicular body subunit 12A<br>CTD-2521M24.9
chr15_+_89182178	4.101	ENST00000559876.1	ISG20	interferon stimulated exonuclease gene 20kDa
chr10_+_92980517	4.077	ENST00000336126.5	PCGF5	polycomb group ring finger 5
chr1_+_110453462	4.041	ENST00000488198.1	CSF1	colony stimulating factor 1 (macrophage)
chr10_-_9801179	4.023	ENST00000419836.1	RP5-1051H14.2	RP5-1051H14.2
chr9_-_80437915	4.005	ENST00000397476.3	GNAQ	guanine nucleotide binding protein (G protein), q polypeptide
chr2_-_231084659	3.978	ENST00000392048.3<br>ENST00000258381.6<br>ENST00000358662.4<br>ENST00000455674.1	SP110<br>SP110	SP110 nuclear body protein<br>SP110 nuclear body protein
chr11_-_4629367	3.972	ENST00000533021.1	TRIM68	tripartite motif containing 68
chr3_-_146262637	3.966	ENST00000472349.1<br>ENST00000342435.4	PLSCR1<br>PLSCR1	phospholipid scramblase 1<br>phospholipid scramblase 1
chr5_-_39203093	3.946	ENST00000515010.1	FYB	FYN binding protein
chr13_+_50070491	3.943	ENST00000442195.1<br>ENST00000496612.1<br>ENST00000357596.3<br>ENST00000485919.1	PHF11<br>PHF11	PHD finger protein 11<br>PHD finger protein 11
chr3_-_146262488	3.918	ENST00000487389.1	PLSCR1	phospholipid scramblase 1

ISMARA

Project

Final-1

Navigation

All motifs sorted by activity significance

All samples sorted alphabetically

Mean activities

Downloads

Activities table

Activity deltas table

Regulatory interactions

Motifs sorted by significance (text)

Download the whole report

chr7_+_134832808	3.895	ENST00000275767.3	TMEM140	transmembrane protein 140
chr6_-_109702885	3.878	ENST00000504373.1	CD164	CD164 molecule, sialomucin
chr2_-_145275828	3.859	ENST00000409211.1<br>ENST00000392861.2	ZEB2<br>ZEB2	zinc finger E-box binding homeobox 2<br>zinc finger E-box binding homeobox 2
chrX_-_30595959	3.859	ENST00000378962.3	CXorf21	chromosome X open reading frame 21
chr2_-_231084820	3.846	ENST00000338556.3<br>ENST00000258382.5	SP110<br>SP110	SP110 nuclear body protein<br>SP110 nuclear body protein
chr3_-_146262365	3.845	ENST00000448787.2	PLSCR1	phospholipid scramblase 1
chr6_+_32811885	3.788	ENST00000458296.1<br>ENST00000413039.1<br>ENST00000429600.1<br>ENST00000412095.1<br>ENST00000415067.1<br>ENST00000395330.1	TAPSAR1<br><br><br><br>TAPSAR1<br>PSMB9	TAP1 and PSMB8 antisense RNA 1<br><br>TAP1 and PSMB8 antisense RNA 1<br>proteasome (prosome, macropain) subunit, beta type, 9
chr5_-_95158375	3.785	ENST00000512469.2<br>ENST00000379979.4<br>ENST00000505427.1<br>ENST00000508780.1	GLRX<br><br><br>GLRX	glutaredoxin (thioltransferase)<br><br>glutaredoxin (thioltransferase)
chr16_+_57023406	3.762	ENST00000436936.1<br>ENST00000262510.6<br>ENST00000308149.7	NLRC5<br>NLRC5	NLR family, CARD domain containing 5<br>NLR family, CARD domain containing 5
chr2_+_33701707	3.758	ENST00000423159.1<br>ENST00000425210.1	RASGRP3<br>RASGRP3	RAS guanyl releasing protein 3 (calcium and DAG-regulated)<br>RAS guanyl releasing prote

Activity graph

Z-values graph

Top targets

GO analysis

Back to top

ISMARA

Project

Final-1

Navigation

All motifs sorted by activity significance

All samples sorted alphabetically

Mean activities

Downloads

Activities table

Activity deltas table

Regulatory interactions

Motifs sorted by significance (text)

Download the whole report

		ENST00000444784.1		in 3 (calcium and DAG-regulated)
chr19_-_50400212	3.749	ENST00000391826.2	IL4I1	interleukin 4 induced 1
chr19_+_17516531	3.701	ENST00000528911.1<br>ENST00000528604.1<br>ENST00000595892.1<br>ENST00000500836.2<br>ENST00000598546.1<br>ENST00000600369.1<br>ENST00000598356.1<br>ENST00000594426.1	MVB12A<br>CTD-2521M24.9	multivesicular body subunit 12A<br>CTD-2521M24.9
chr6_-_36355486	3.624	ENST00000538992.1	ETV7	ets variant 7
chr13_+_50070077	3.601	ENST00000426879.1<br>ENST00000378319.3	PHF11<br>PHF11	PHD finger protein 11<br>PHD finger protein 11
chr14_-_67981870	3.573	ENST00000555994.1	TMEM229B	transmembrane protein 229B
chr6_-_36355513	3.569	ENST00000373737.4<br>ENST00000340181.4	ETV7<br>ETV7	ets variant 7<br>ets variant 7
chr15_+_89181974	3.557	ENST00000306072.5	ISG20	interferon stimulated exonuclease gene 20kDa
chr7_+_150382781	3.555	ENST00000474605.1<br>ENST00000223293.5	GIMAP2<br>GIMAP2	GTPase, IMAP family member 2<br>GTPase, IMAP family member 2

Gene Ontology Analysis

Activity graph

Z-values graph

Top targets

GO analysis

Back to top

Gene overrepresentation in biological_process category:

Search:

Show 10 entries

Showing 1 to 20 of 209 entries

ISMARA

Project

Final-1

Navigation

All motifs sorted by activity significance

All samples sorted alphabetically

Mean activities

Downloads

Activities table

Activity deltas table

Regulatory interactions

Motifs sorted by significance (text)

Download the whole report

Log-likelihood per target	Total log-likelihood	Term	Description
5.3	628.3	GO:0071357	type I interferon signaling pathway(GO:
1.1	131.0	GO:0051607	defense response to virus(GO:005160
0.1	117.2	GO:0006955	immune response(GO:0006955)
1.1	73.2	GO:0032480	negative regulation of type I interferon
1.1	51.0	GO:0042130	negative regulation of T cell proliferatio
11.0	44.1	GO:0051097	negative regulation of helicase activity(
0.1	38.2	GO:0007249	I-kappaB kinase/NF-kappaB signaling(
9.5	38.1	GO:0009138	pyrimidine nucleoside diphosphate me
0.2	36.8	GO:0042787	protein ubiquitination involved in ubiqui
2.4	29.2	GO:0002230	positive regulation of defense response
0.2	29.0	GO:0009615	response to virus(GO:0009615)
0.6	26.3	GO:0060135	maternal process involved in female pr
4.1	24.3	GO:0006436	tryptophanyl-tRNA aminoacylation(GO:
1.9	24.2	GO:0017121	phospholipid scrambling(GO:0017121)
7.3	22.0	GO:0032938	negative regulation of translation in res
0.3	20.8	GO:0060333	interferon-gamma-mediated signaling p
0.1	20.4	GO:0007281	germ cell development(GO:0007281)
0.5	19.1	GO:0070206	protein trimerization(GO:0070206)
4.7	19.0	GO:0046967	cytosol to ER transport(GO:0046967)
4.7	18.8	GO:0035279	mRNA cleavage involved in gene silen

Gene overrepresentation in cellular_component category:

Search:

Show 10 entries

Showing 1 to 20 of 86 entries

Log-likelihood per target	Total log-likelihood	Term	Description
0.1	289.0	GO:0005829	cytosol(GO:0005829)
0.0	239.2	GO:0005575	cellular_component(GO:0005575)
0.0	168.0	GO:0005737	cytoplasm(GO:0005737)

Activity graph

Z-values graph

Top targets

GO analysis

Back to top

ISMARA

Project

Final-1

Navigation

All motifs sorted by activity significance

All samples sorted alphabetically

Mean activities

Downloads

Activities table

Activity deltas table

Regulatory interactions

Motifs sorted by significance (text)

Download the whole report

0.4	162.1	GO:0005792	obsolete microsome(GO:0005792)
0.1	152.8	GO:0005739	mitochondrion(GO:0005739)
0.2	135.8	GO:0005789	endoplasmic reticulum membrane(GO:
0.1	58.5	GO:0010008	endosome membrane(GO:0010008)
0.1	53.0	GO:0048471	perinuclear region of cytoplasm(GO:00
0.0	52.7	GO:0005730	nucleolus(GO:0005730)
0.0	27.8	GO:0005615	extracellular space(GO:0005615)
2.6	23.8	GO:0042825	TAP complex(GO:0042825)
0.5	22.3	GO:0012505	endomembrane system(GO:0012505)
7.3	22.0	GO:0042406	extrinsic component of endoplasmic ret
0.2	21.7	GO:0016605	PML body(GO:0016605)
1.7	21.5	GO:0033391	chromatoid body(GO:0033391)
0.3	19.8	GO:0015030	Cajal body(GO:0015030)
0.3	19.2	GO:0000932	cytoplasmic mRNA processing body(G
0.1	16.6	GO:0016363	nuclear matrix(GO:0016363)
0.1	16.5	GO:0009897	external side of plasma membrane(GO
1.6	14.3	GO:0008074	guanylate cyclase complex, soluble(GC

Gene overrepresentation in molecular_function category:

Search:
Show 10 entries
Showing 1 to 20 of 182 entries

Log-likelihood per target	Total log-likelihood	Term	Description
0.9	163.3	GO:0016779	nucleotidyltransferase activity(GO:0016
0.6	134.1	GO:0004386	helicase activity(GO:0004386)
1.5	122.4	GO:0051536	iron-sulfur cluster binding(GO:0051536
0.2	97.4	GO:0003924	GTPase activity(GO:0003924)
0.0	92.1	GO:0008270	zinc ion binding(GO:0008270)
0.4	79.5	GO:0016881	acid-amino acid ligase activity(GO:001
1.6	73.3	GO:0003950	NAD+ ADP-ribosyltransferase activity(

Activity graph

Z-values graph

Top targets

GO analysis

Back to top

ISMARA

Project

Final-1

Navigation

All motifs sorted by activity significance

All samples sorted alphabetically

Mean activities

Downloads

Activities table

Activity deltas table

Regulatory interactions

Motifs sorted by significance (text)

Download the whole report

0.6	40.8	GO:0036459	thiol-dependent ubiquitin-specific prote
13.4	40.1	GO:0031386	protein tag(GO:0031386)
5.4	38.1	GO:0004127	cytidylate kinase activity(GO:0004127)
0.3	31.2	GO:0003704	obsolete specific RNA polymerase II tra
0.1	29.6	GO:0043565	sequence-specific DNA binding(GO:00
5.7	28.6	GO:0003726	double-stranded RNA adenosine deam
1.4	25.6	GO:0008035	high-density lipoprotein particle binding
0.1	24.7	GO:0003714	transcription corepressor activity(GO:0
4.1	24.3	GO:0004830	tryptophan-tRNA ligase activity(GO:000
1.9	24.2	GO:0017128	phospholipid scramblase activity(GO:0
0.0	23.5	GO:0004872	receptor activity(GO:0004872) molecul
0.5	22.5	GO:0008603	cAMP-dependent protein kinase regula
5.5	22.0	GO:0032183	SUMO binding(GO:0032183)

Gene overrepresentation in C2:CP category:

Search:

Show 10 entries

Showing 1 to 18 of 18 entries

Log-likelihood per target	Total log-likelihood	Term
2.4	28.3	ST_INTERFERON_GAMMA_PATHWAY
0.1	23.3	SIG_PIP3_SIGNALING_IN_CARDIAC_MYOCTES
0.6	21.6	SA_MMP_CYTOKINE_CONNECTION
0.4	11.8	SIG_PIP3_SIGNALING_IN_B_LYMPHOCYTES
0.3	10.5	SA_FAS_SIGNALING
0.4	7.0	SIG_IL4RECEPTOR_IN_B_LYPHOCYTES
0.3	6.7	ST_INTERLEUKIN_4_PATHWAY
0.2	5.7	SA_CASPASE_CASCADE
0.7	5.2	ST_TYPE_I_INTERFERON_PATHWAY
0.3	4.4	SA_PTEN_PATHWAY
0.3	3.4	ST_PAC1_RECEPTOR_PATHWAY

Activity graph

Z-values graph

Top targets

GO analysis

Back to top

0.1	2.5	ST_ADRENERGIC
0.0	1.8	SIG_REGULATION_OF_THE_ACTIN_CYTOSKELET
0.0	1.6	ST_WNT_BETA_CATENIN_PATHWAY
0.0	0.8	ST_P38_MAPK_PATHWAY
0.1	0.7	ST_JAK_STAT_PATHWAY
0.0	0.6	SIG_INSULIN_RECEPTOR_PATHWAY_IN_CARDIAC
0.0	0.3	SA_PROGRAMMED_CELL_DEATH

ISMARA

Project

Final-1

Navigation

All motifs sorted by activity significance

All samples sorted alphabetically

Mean activities

Downloads

Activities table

Activity deltas table

Regulatory interactions

Motifs sorted by significance (text)

Download the whole report

SIB Swiss Institute of Bioinformatics

BIOZENTRUM Universität Basel The Center for Molecular Life Sciences

van Nimwegen Lab

Activity graph

Z-values graph

Top targets

GO analysis

Back to top
